# Association of computed tomography‐derived body composition and complications after colorectal cancer surgery: A systematic review and meta‐analysis

**DOI:** 10.1002/jcsm.13580

**Published:** 2024-10-06

**Authors:** Claire P.M. van Helsdingen, Job G.A. van Wijlick, Ralph de Vries, Nicole D. Bouvy, Mariska M.G. Leeflang, Robert Hemke, Joep P.M. Derikx

**Affiliations:** ^1^ Department of Pediatric Surgery, Emma Children's Hospital Amsterdam UMC, location University of Amsterdam Amsterdam The Netherlands; ^2^ Tytgat Institute for Liver and Intestinal Research Amsterdam UMC, location University of Amsterdam Amsterdam The Netherlands; ^3^ Amsterdam Gastroenterology Endocrinology Metabolism Amsterdam The Netherlands; ^4^ Medical Library Vrije Universiteit Amsterdam Amsterdam The Netherlands; ^5^ Department of Surgery Maastricht University Medical Center Maastricht The Netherlands; ^6^ NUTRIM School of Nutrition and Translational Research in Metabolism Maastricht University Medical Center Maastricht The Netherlands; ^7^ Department of Epidemiology and Data Science Amsterdam UMC, location University of Amsterdam Amsterdam The Netherlands; ^8^ Department of Radiology and Nuclear Medicine Amsterdam UMC, location University of Amsterdam Amsterdam The Netherlands

**Keywords:** adipose tissue, body composition, colorectal cancer, colorectal surgery complications, computed tomography, muscle tissue

## Abstract

The prediction of the risk of developing complications after colorectal surgery for colorectal carcinoma remains imprecise. Body composition measurements on a computed tomography (CT) scan can potentially contribute to a better preoperative risk assessment. The aim of this systematic review is to evaluate the evidence for the use of body composition measurements on CT scans to predict short‐term complications after colorectal cancer surgery. A literature search (in PubMed, Embase and Web of Science) was performed up to 1 August 2022. Two researchers independently screened the articles, extracted data and assessed the quality of the studies using the Quality in Prognosis Studies tool. The primary outcome measure was the occurrence of complications within 30 days after surgery. Meta‐analysis was conducted using a random‐effects model to synthesize a pooled odds ratio (OR). The study protocol was registered in PROSPERO (CRD42021281010). Forty‐five articles with a total of 16 537 patients were included. In total, 26 body composition measures were investigated: 8 muscle‐related measures, 11 adipose tissue measures, 4 combined muscle and adipose tissue measures, and 3 other measures. These were investigated as potential predictors for more than 50 differently defined postoperative complications. Meta‐analysis was only possible for two measurements and showed that higher amounts of visceral fat increase the risk of developing overall complications (OR: 2.52 [1.58–4.00], *P* < 0.0001) and anastomotic leakage (OR: 1.76 [1.17–2.65], *P* = 0.006). A wide variety of body composition measurements on preoperative CT scans have been investigated as a predictive factor for postoperative complications. Visceral fat appeared to be associated with overall complications and anastomotic leakage; however, the association is weak, and its clinical relevance or applicability is questionable. The current evidence is limited by methodological heterogeneity and the risk of bias. To improve comparability of results across studies and improve decision‐making, future studies should use standardized methods for measuring body composition on CT scans, outcome definitions and statistical analyses.

## Introduction

The number of patients with colorectal carcinoma (CRC) has unabatedly been increasing, and survival from CRC has generally been increasing as well, partly due to improved national surveillance strategies.^1^, ^2^, ^3^, ^4^ Surgical resection of the tumour is a cornerstone of the treatment of CRC, which comes with the risk of developing postoperative complications. The most common complications after CRC surgery are anastomotic leakage (AL), surgical site infections (SSIs) and postoperative ileus (POI), with incidences ranging between 5–20%, 1–9% and 3–32%, respectively.^5^, ^6^, ^7^ When complications occur, morbidity rates, long‐term mortality rates, duration of hospital stay and costs rise and quality of life decreases.^8^, ^9^, ^10^ Therefore, it is of great value to predict which patient is going to develop complications, as it will aid surgeons in the preoperative decision‐making process for the optimal surgical strategy to decrease the chance of complications. Despite having identified several risk factors for developing surgical complications, it remains difficult for surgeons to preoperatively assess the risk of postoperative complications. In recent years, the impact of the body composition (BC) of the patient on the postoperative course has been gaining more attention, and interventional programmes are developed to improve the preoperative condition.^11^ A sarcopenic state, which is characterized by loss of muscle mass, strength and performance, is associated with increased length of hospital stay, increased number of major complications, increased morbidity and decreased overall survival after various types of oncological surgery.^12^, ^13^, ^14^, ^15^ In addition to muscle‐associated disorders, the role of adipose tissue (AT), both visceral and subcutaneous, on postoperative outcomes is investigated as well, which seems to have a negative effect on the perioperative course.^16^, ^17^ Therefore, assessing BC can be of use for preoperative risk assessment of patients who need to undergo surgery for CRC. Most BC measurements are performed on computed tomography (CT) scans. As CT scans are part of the clinical work‐up of patients with CRC, it is relatively easy to assess BC and use these measurements for preoperative risk assessment.^18^ BC measurements obtained from CT scans are increasingly being studied as potential predictors of postoperative outcomes, such as surgical and medical complications, in CRC patients. However, the results of these studies are ambiguous. This ambiguity may be caused by differences in investigated patient populations, but it is likely mainly due to differences in methods of CT measurements and further analyses. Hence, it is important to gain insight into how BC measurements are performed, analysed and reported. Thus, the aim of this systematic review (SR) is to comprehensively evaluate the evidence for the use of BC measurements on CT scans to predict short‐term (within 30 days postoperative) complications after CRC surgery.

## Methods

### Literature review

This review is reported according to the Preferred Reporting Items for Systematic Reviews and Meta‐Analyses (PRISMA) (https://www.prisma‐statement.org/).^19^ The research protocol was registered on PROSPERO (Identification Number CRD42021281010).

### Search strategy

To identify all relevant publications, we conducted systematic searches in the bibliographic databases MEDLINE (through PubMed), Embase (via embase.com) and Web of Science (Core Collection) from inception to 1 August 2022. The following terms were used (including synonyms and closely related words) as index terms or free‐text words: ‘Colorectal Neoplasms’, ‘Body Composition’, ‘Colorectal Surgery’ and ‘X‐Ray Computed Tomography’. The references of the identified articles were searched for relevant publications. All languages were accepted. Duplicate articles were excluded by a medical information specialist using Endnote X20.0.1 (Clarivate™), following the Amsterdam Efficient Deduplication (AED) method and the Bramer method.^20^, ^21^ The full search strategy can be found in *Table* *S1*.

### Selection process

Two reviewers (CPMvH and JGAvW) independently screened all potentially relevant titles and abstracts for eligibility using the web application Rayyan.^22^ If necessary, the full‐text article was checked for the eligibility criteria. Differences in judgement were resolved through a consensus procedure; if needed, an independent third reviewer (JPMD) was consulted. Studies were included if they met the following criteria: patients aged ≥18 years who underwent colorectal surgery for CRC; preoperative BC measurement performed on CT; reported complications within 30 days postoperatively; and assessed the BC measurement as a prognostic value on the outcome. Publication types, such as editorials, conference abstracts, letters, legal cases and interviews, were excluded.

### Outcomes

The primary outcome of this study was postoperative complications ≤30 days after the operation. Postoperative complications were defined as all complications described in the studies, both surgical complications, such as POI, SSI and AL, and medical complications, for example, pneumonia, urinary tract infection (UTI) and neurological complications. Also, combinations of different postoperative complications graded using severity grading scales such as the Clavien–Dindo (CD) classification were defined as postoperative outcomes^23^ (see *Table* *1* for an overview of the definitions).

**Table 1:** Study characteristics

Study	Year	Study design	No. of centres	Country	Sample size (*N* total)	Groups (*N*)	Age (years), mean ± SD/[range], median (IQR)/[range] or *N* (%)	Male, *N* (%)	BMI, mean ± SD/[range], median (IQR)/[range] or *N* (%)	Tumour location, *N* (%)	Surgical approach, *N* (%)	Neoadjuvant treatment, *N* (%)	Time of follow‐up	Outcomes	Severity measurement	Definition of complication
Baastrup et al.^24^	2019	RS	1	Denmark	102	1. VO (26) 2. No‐VO (76)	1. 67.1 ± 10.1^a^ 2. 64.0 ± 11.5^a^	1. 23 (88.5) 2. 41 (53.9)	1. 29.8 ± 3.4^a^ 2. 24.4 ± 3.3^a^	All rectum	Laparoscopy, 102 (100)	‐	30 days postoperative	1. AL 2. Total complications 3. Severe complications	Clavien–Dindo classification	Anastomotic leak: Rahbari et al. grade B of C Total: CD ≥ 1 Severe: CD ≥ 3
Bachmann et al.^25^	2018	RS	1	Belgium	90	‐	67 (13)^a^ 68 [41–89]^b^	54 (60)	‐	Colon, 49 (54) Rectum, 41 (46)	Open, 34 (38) Laparoscopic, 50 (55) Conversion, 6 (7)	‐	30 days postoperative	1. Overall complications 2. Severe complications	Clavien–Dindo classification	Severe: CD ≥ 3
Ballian et al.^26^	2012	RS	1	The United States	113	‐	59 ± 13 [33–89]^a^	67 (59)	27.6 ± 6.3 [15.9–50.0]^a^	All rectum	Laparoscopic, 18 (16) Open, 95 (84)	Radiotherapy, 84 (74)	21 days postoperative	Complication grade	Mazeh et al.	‐
Boer et al.^27^	2016	RS	1	The Netherlands	91	‐	71.3 ± 9.74^a^	49 (54)	27.0 ± 4.09^a^	Left‐sided, 59 (65) Right sided, 32 (35)	Open, 91 (100)	None, exclusion criterion	30 days of surgery	1. Overall complications 2. Severe postoperative complications	Clavien–Dindo classification	Overall: CD ≥ 1 Severe complications: CD ≥ 3a
Cakir et al.^28^	2015	RS	1	The Netherlands	564	1. Total 2. VO (367) 3. No‐VO (197)	1. 70 ± 11^a^ 2. 71 ± 10^a^ 3. 68 ± 12^a^	1. 287 (51) 2. 237 (65) 3. 50 (25)	25.6^a^	All colon	Open and laparoscopic	‐	30 days after surgery	1. AL 2. Pneumonia 3. Wound infection 4. UTI	‐	‐
Chai et al.^29^	2021	RS	1	Australia	228	1. Total 2. No‐SA (192) 3. SA (36)	1. 69^b^ 2. 67.5 [58–77]^b^ 3. 78 [68.25–82.75]^b^	1. 139 (61) 2. 115 (60) 3. 24 (67)	1. 27.93^b^ 2. 28.10 [23.89–32.46]^b^ 3. 27.41 [23.72–30.37]^b^	1. Colon, 101 (44) Rectum, 127 (56) 2. Colon, 82 (43) Rectum, 110 (57) 3. Colon, 19 (53) Rectum, 17 (47)	1. Open, 32 (14) 2. Open, 20 (10) 3. Open, 12 (33)	‐	30 days after surgery	1. Overall complications 2. Major complications 3. Anastomotic leak 4. Wound infection 5. Respiratory complication 6. Cardiac complication 7. Renal complication 8. Gastrointestinal complication 9. Mortality	Clavien–Dindo classification	Overall complications: CD ≥ 2 Major complications: CD ≥ 3 Anastomotic leak: Diagnosed either radiologically or surgically
Chen et al.^30^	2018	PS	1	China	376	1. Total 2. No‐SA and no‐VO (134) 3. SA and no‐VO (51) 4. No‐SA and VO (150) 5. SA and VO (41)	1. 64.33 ± 12.3^a^ 2. 59.2 ± 13.0^a^ 3. 70.73 ± 12.61^a^ 4. 64.83 ± 9.92^a^ 5. 71.29 ± 10.24^a^	1. 228 (61) 2. 90 (67) 3. 34 (67) 4. 94 (63) 5. 10 (24)	1. 23.02 ± 3.10^a^ 2. 21.84 ± 2.38^a^ 3. 20.50 ± 2.85^a^ 4. 25.01 ± 2.63^a^ 5. 22.70 ± 2.78^a^	1. Colon, 210 (56) Rectum, 166 (44) 2. Colon, 74 (55) Rectum, 60 (45) 3. Colon, 29 (57) Rectum, 22 (43) 4. Colon, 83 (55) Rectum, 67 (45) 5. Colon, 24 (59) Rectum, 17 (41)	1. Laparoscopic, 149 (40) 2. Laparoscopic, 59 (44) 3. Laparoscopic, 16 (31) 4. Laparoscopic, 60 (40) 5. Laparoscopic, 14 (34)	None, exclusion criterion	30 days after surgery	1. Total complications 2. Surgical complications 3. Medical complications	Clavien–Dindo classification	Total postoperative: CD ≥ 2 Surgical: Gastrointestinal dysfunction, wound infection, bleeding, intra‐abdominal abscess, AL and intestinal obstruction Medical: Pulmonary complications, cardiac complications, venous thrombosis, persistent hypoalbuminaemia and UTI
der Hagopian et al.^31^	2018	RS	1	Sweden	605	1. Total 2. PRF > 40 (172) 3. PRF < 40 (433)	1. 72 (65–79)^b^ 2. 72 (65–78)^b^ 3. 73 (64–80)^b^	1. 297 (49) 2. 148 (86) 3. 149 (34)	1. 25 (23–29)^b^ 2. 29 (26–32)^b^ 3. 24 (22–27)^b^	All colon	1. Laparoscopic, 153 (25) Open, 452 (75) 2. Laparoscopic, 39 (23) Open, 133 (77) 3. Laparoscopic, 114 (26) Open, 319 (74)	‐	30 days after surgery	1. Any complication 2. CD grade (I, II, III, IV and V) 3. Surgical complications 4. SSI 5. Intra‐abdominal infection 6. Wound dehiscence 7. AL 8. Infectious 9. Cardiovascular	Clavien–Dindo classification	Any complication: CD ≥ 1 Surgical: SSI, intra‐abdominal infection, AL and wound dehiscence
Dong et al.^32^	2022	RS	1	China	528	1. Total 2. VO (261) 3. No‐VO (267)	1. 74.1 ± 6.2.3^a^ 2. 74.0 ± 6.0^a^ 3. 74.1 ± 6.4^a^	1. 318 (60) 2. 138 (53) 3. 180 (67)	1. 22.5 ± 3.1^a^ 2. 24.2 ± 2.7^a^ 3. 20.9 ± 2.5^a^	1. Colon, 285 (54) Rectum, 243 (46) 2. Colon, 148 (57) Rectum, 113 (43) 3. Colon, 137 (51) Rectum, 130 (49)	1. Laparoscopic, 235 (45) 2. Laparoscopic, 124 (48) 3. Laparoscopic, 111 (42)	None, exclusion criterion	During hospital stay or within 30 days after operation	1. Total complications 2. Surgical complications 3. Gastrointestinal dysfunction 4. Wound infection 5. Bleeding 6. Intra‐abdominal abscess 7. AL 8. Intestinal obstruction 9. Medical complications 10. Pulmonary complications 11. Cardiac complications 12. Venous thrombosis 13. Persistent hypoalbuminaemia 14. UTI	Clavien–Dindo classification	Postoperative complications: CD ≥ 2 Surgical complications: Gastrointestinal dysfunction, wound infection, bleeding, intra‐abdominal abscess, AL and intestinal obstruction Medical complications: Pulmonary complications, cardiac complications, venous thrombosis, persistent hypoalbuminaemia and UTI
Frostberg et al.^33^	2021	RS	1	Denmark	278	1. Total 2. No‐VO (148) 3. VO (130) 4. Low SAT (70) 5. Moderate SAT (138) 6. High SAT (70)	1. 70^b^ 2. 70^b^ 3. 71.5^b^ 4. 72.5^b^ 5. 70^b^ 6. 70^b^	1. 165 (60) 2. 65 (44) 3. 100 (77) 4. 46 (66) 5. 88 (64) 6. 31 (44)	1. 25.2^b^ 2. 23.2^b^ 3. 27.4^b^ 4. 22.5^b^ 5. 25.2^b^ 6. 28.4^b^	1. Colon, 175 (63) Rectum, 103 (37) 2. Colon, 93 (63) Rectum, 55 (37) 3. Colon, 82 (63) Rectum, 48 (37) 4. Colon, 40 (57) Rectum, 30 (43) 5. Colon, 87 (63) Rectum, 51 (37) 6. Colon, 48 (69) Rectum, 22 (31)	1. Open, 168 (60) Laparoscopic, 110 (40) 2. Open, 88 (60) Laparoscopic, 60 (40) 3. Open, 80 (65) Laparoscopic, 50 (35) 4. Open, 46 (66) Laparoscopic, 24 (34) 5. Open, 83 (60) Laparoscopic, 55 (40) 6. Open, 39 (56) Laparoscopic, 31 (44)	‐	30 days after surgery	Overall complications	‐	Surgical complications: Intraoperative lesions, ileus, bleeding, superficial wound infection, intra‐abdominal infection, AL and burst abdomen Medical complications: Thrombo‐embolic events (stroke, acute myocardial infarction, venous thrombo‐embolism and arterial embolism), aspiration pneumonia, heart and/or lung and/or kidney failure, and sepsis
Hanaoka et al.^34^	2017	RS	1	Japan	133	1. Total 2. Mild MPM (105) 3. Severe MPM (28)	1. 68.3 ± 9.8^a^ 2. 67.1 ± 9.6^a^ 3. 73.1 ± 9.5^a^	1. 84 (63) 2. 68 (65) 3. 16 (57)	1. 21.4 ± 2.8^a^ 2. 21.4 ± 2.7^a^ 3. 21.1 ± 3.2^a^	1. Colon, 87 (65) Rectum, 46 (35) 2. Colon, 68 (65) Rectum, 37 (35) 3. Colon, 19 (68) Rectum, 9 (32)	1. Laparoscopic, 88 (66) Open, 45 (34) 2. Laparoscopic, 73 (70) Open, 32 (30) 3. Laparoscopic, 15 (54) Open, 13 (46)	None	30 days after surgery	1. Overall complications 2. Infectious complications 3. Non‐infectious complications	Clavien–Dindo classification	Overall: CD ≥ 1 Infectious: Wound infection, intra‐abdominal abscess, anastomotic leak, pneumonia and septicaemia CD ≥ 1 Non‐infectious: Bowel obstruction, acute onset atrial fibrillation, TIA, anastomosis haemorrhage, lymphorrhea, neurogenic bladder dysfunction and choledocholithiasis Wound infection: Presence of pus, either discharged spontaneously or requiring drainage Intra‐abdominal abscess: By either surgical drainage or ultrasonographically guided aspiration of pus AL: Radiologically verified fistula to bowel anastomosis or diagnosed by re‐laparotomy Pneumonia: Fever above 38.5°C, a positive X‐ray and the requirement for antibiotic treatment Septicaemia: Clinical symptoms combined with a positive blood culture
He et al.^35^	2021	RS	1	China	129	‐	66 (60.0–73.5)^b^	70 (54)	23.8 (21.7–26.0)^b^	Colon, 83 (63) Rectum, 48 (37)	All laparoscopic	None, exclusion criterion	30 days after surgery	Overall complications	‐	Overall complications: Wound infection, pneumonia, intra‐abdominal infection, AL and ileus
Heus et al.^36^	2019	RS	1	The Netherlands	406	1. Total 2. VFA < 100 (134) 3. VFA > 100 (272)	1. 67 ± 11^a^ 2. 65 ± 12^a^ 3. 69 ± 9^a^	1. 253 (62) 2. 61 (46) 3. 192 (71)	1. 25.5 ± 3.5^a^ 2. 23.5 ± 2.8^a^ 3. 26.6 ± 3.5^a^	All rectum	1. Laparoscopic, 103 (25) Conversion, 9 (9) 2. Laparoscopic, 30 (21) Conversion, 3 (10) 3. Laparoscopic, 73 (27) Conversion, 6 (8)	1. Radiotherapy, 263 (65) Chemoradiotherapy, 107 (26) 2. Radiotherapy, 87 (65) Chemoradiotherapy, 38 (28) 3. Radiotherapy, 176 (65) Chemoradiotherapy, 69 (25)	In‐hospital	1. Overall complications 2. Wound infection 3. AL 4. Ileus 5. UTI 6. Pneumonia 7. CD1 8. CD2 9. CD3 10. CD4 11. Mortality	Clavien–Dindo classification	Overall: Wound infection, pneumonia, UTI, AL and ileus
Heus et al.^37^	2016	RS	1	The Netherlands	74	1. Total 2. VFA < 100 (30) 3. VFA > 100 (44) 4. VFA < 130 (43) 5. VFA > 130 (31)	1. 64 ± 10.0^a^ 2. 61.2 ± 8.0^a^ 3. 66.0 ± 11.8^a^ 4. 61 ± 11^a^ 5. 68 ± 7^a^	1. 39 (53) 2. 9 (30) 3. 30 (68) 4. 20 (47) 5. 19 (61)	‐	All rectum	1. Laparoscopic, 15 (21) 2. Laparoscopic, 5 (17) 3. Laparoscopic, 10 (23) 4. Laparoscopic, 7 (17) 5. Laparoscopic, 8 (26)	All chemoradiation	30 days after surgery	1. Overall complications 2. Wound infection 3. Pneumonia 4. UTI 5. AL 6. Ileus 7. Mortality	‐	Overall: Wound infection, pneumonia, UTI, AL and ileus
Jochum et al.^38^	2019	RS	1	The United States	47	1. Total 2. SA (24) 3. No‐SA (24)	1. 59.3 ± 11.4 [36–82]^a^ 2. 60 ± 10.9^a^ 3. 59 ± 11.7^a^	1. 29 (62) 2. 16 (67) 3. 13 (57)	1. 29.2 ± 5.6 [19.0–43.4]^a^	All rectum	‐	All chemoradiation	30 days after surgery	Overall complications	‐	‐
Kuritzkes et al.^39^	2018	RS	1	The United States	266	‐	67.5 (56–77)^b^	139 (53)	26.9 (23.8–30.5)^b^	All colon	Laparoscopic, 189 (71)	None, exclusion criterion	30 days after surgery	Major morbidity	Clavien–Dindo classification	Major morbidity: CD ≥ 3
Lieffers et al.^40^	2012	RS	1	Canada	234	1. Total 2. No‐SA (142) 3. SA (91)	1. 63 ± 12^a^ 2. 61 ± 11^a^ 3. 66 ± 12^a^	1. 135 (58) 2. 78 (55) 3. 57 (63)	1. 28.5 ± 5.3^a^ 2. 30.0 ± 5.4^a^ 3. 26.1 ± 4.2^a^	1. Colon, 137 (59) Rectosigmoid, 27 (11) Rectum, 70 (30) 2. Colon, 83 (58) Rectosigmoid, 16 (11) Rectum, 44 (31) 3. Colon, 54 (59) Rectosigmoid, 11 (12) Rectum, 26 (29)	1. Laparoscopic, 18 (8) 2. Laparoscopic, 12 (8) 3. Laparoscopic, 6 (7)	‐	30 days after discharge from index surgical admission	Postoperative infections	‐	Postoperative infections: ICD‐10 diagnostic codes (the following ICD‐10 diagnostic codes related to infection were found: UTI [N39.0], pneumonia [J18.X], peritonitis (K65.0 and K65.8), septicaemia [A41.X], infection following a procedure [T81.4], enterocolitis due to *Clostridium difficile* [A04.7], and diarrhoea and gastroenteritis of presumed infectious origin [A09])
Liu et al.^41^	2019	RS	1	China	374	1. SSI (73) 2. No‐SSI (301)	1. 68.3 ± 7.8^a^ 2. 60.4 ± 8.3^a^	1. 49 (67) 2. 191 (63)	1. 25.6 ± 3.9^a^ 2. 22.3 ± 2.9^a^	All colon	All open	‐	30 days after surgery	SSI	‐	SSI: An infection that appeared at the surgical site within 30 postoperative days and that was characterized by any of the following circumstance: purulent drainage from surgical site, organism cultured from the fluid of surgical site and/or incisional inflammation (pain, tenderness, localized swelling and redness). All three subtypes of SSI including superficial, deep and organ/space SSI were taken into account for subsequent analysis. Definition is according to the WHO criteria
Looijaard et al.^42^	2020	RS	1	The Netherlands	254	‐	73.6 (69.7–78.5)^b^	158 (62.2)	27.4 ± 4.0^a^	All colon	Laparoscopic, 169 (67)	‐	During admission or within 30 days after surgery	Severe complications	Clavien–Dindo classification	Severe: CD ≥ 3 surgical of medical complications
Looijaard et al.^43^	2019	RS	1	The Netherlands	378	‐	73.4 (69.5–78.4)^b^	228 (60)	27.1 ± 4.1^a^	Colon, 284 (75) Rectum, 94 (25)	Laparoscopic, 217 (57)	‐	During admission or within 30 days after surgery	Severe complications	Clavien–Dindo classification	Severe: CD ≥ 3 Surgical complications included AL, intra‐abdominal abscess, wound or fascial dehiscence, stoma‐related complications, blood loss >500 mL or postoperative haemorrhage, incisional hernia, bowel injury or injury to another organ. Medical complications included respiratory, cardiac, thrombo‐embolic, infectious, neurological and other complications
Margadant et al.^44^	2016	RS	1	The Netherlands	373	1. Total 2. Low muscle density (92) 3. High muscle density (281)	1. 78 (75–82)^b^ 2. 79 (79–83)^b^ 3. 77 (74–82)^b^	1. 181 (43) 2. 46 (50) 3. 135 (48)	1. 25 (23–28)^b^ 2. 26 (23–30)^b^ 3. 25 (23–28)^b^	1. Colon, 287 (77) Rectum, 86 (23) 2. Colon, 74 (80) Rectum, 18 (20) 3. Colon, 213 (76) Rectum, 68 (24)	1. Laparoscopic, 171 (46) Open, 194 (52) 2. Laparoscopic, 35 (38) Open, 55 (60) 3. Laparoscopic, 136 (48) Open, 139 (49)	‐	30 days after surgery	1. Major complications 2. Mortality 3. AL	Clavien–Dindo classification	Major complications: CD ≥ 3
Martin et al.^45^	2018	RS	2	Canada and the United Kingdom	2100	‐	66.6 ± 11.9^a^	1270 (61)	27.7 ± 5.6^a^	Colon, 1279 (61) Rectum, 821 (39)	Laparoscopic, 1580 (75) Open, 413 (20) Converted, 83 (4) Unknown, 24 (1)	‐	30 days after surgery	Major complications	Clavien–Dindo classification	Major complications: CD ≥ 3
Maurício et al.^46^	2018	PS	1	Brazil	84	‐	61.6 ± 13.1^a^	39 (46)	‐	Colon, 48 (57) Rectum, 36 (42)	Laparoscopic, 39 (46) Open, 45 (53)	33 (39)	Until discharge (median 8 [IQR 7–12] days)	Complications	Clavien–Dindo classification	Complications: CD ≥ 2
Mizuuchi et al.^47^	2022	RS	1	Japan	197	‐	65.2 [26–87]^b^	‐	22.6 [15.9–31.9]^b^	Rectosigmoid, 53 (27) Upper rectum, 65 (33) Lower rectum, 79 (40)	Laparoscopic, 156 (79)	None, exclusion criterion	30 days after surgery or before discharge	AL	Clavien–Dindo classification	AL: Stool discharge from the intra‐abdominal drain, positive bacterial culture from the intra‐abdominal drain, radiological evidence of peritonitis or intra‐abdominal abscess by CT scan, or enema with rectal contrast and CD > 2
Morimoto et al.^48^	2019	RS	1	Japan	417	‐	64.9 ± 12.9^a^	259 (62)	22.5 ± 3.4^a^	Colon, 245 (59) Rectum, 172 (41)	Laparoscopic, 378 (91)	19 (5)	30 days after surgery	Ileus	Clavien–Dindo classification	Ileus: Patients complained of nausea, vomiting or abdominal distension, and dilatation of the small bowel was radiologically confirmed without obvious SBO and CD ≥ 2
Nakamura et al.^49^	2022	PS	1	Japan	474	‐	≥70 years: 245 (52%)	263 (56)	≥22 kg/m^2^: 259 (55%)	Right colon, 165 (36)	Laparoscopic, 403 (87)	‐	30 days after surgery	EPSBO	Clavien–Dindo classification	EPSBO: Imaging findings of dilation of small intestine and air fluid levels in abdominal X‐ray or CT with three or more physical symptoms (abdominal pain, abdominal fullness, nausea, vomiting, no defecation and flatus more than 24 h) or findings (metallic sound, hyperperistalsis and abdominal tenderness)
Nakanishi et al.^50^	2018	RS	1	Japan	494	1. Total 2. No‐SA (196) 3. SA (298)	1. 66.1 ± 12.4 2. 65.4 ± 12.6 3. 66.5 ± 12.1	1. 298 (60) 2. 110 (56) 3. 188 (71)	1. 22.2 ± 3.6 2. 24.1 ± 3.5 3. 21.0 ± 3.2	1. Colon, 284 (58) Rectum, 210 (42) 2. Colon, 117 (60) Rectum, 79 (40) 3. Colon, 167 (56) Rectum, 131 (44)	1. Laparoscopic, 262 (53) 2. Laparoscopic, 106 (54) 3. Laparoscopic, 156 (53)	‐	Between day of surgery and day of discharge, 18.3 ± 15.9 POD	1. Complication CD ≥ 1 2. Complication CD ≥ 2 3. Complication CD > 2 4. Complication CD > 3 5. Incisional SSIs 6. Intra‐abdominal abscesses 7. Infections other than surgical site (pneumonia, cholecystitis and UTI) 8. AL 9. Ileus, haemorrhaging and other complications (acute heart failure, cardiac arrhythmia, brain infarction, atelectasis and gastric ulcer)	Clavien–Dindo classification	Complication: Incisional SSIs, intra‐abdominal abscesses, infections other than surgical site (such as pneumonia, cholecystitis and UTI), AL, ileus, haemorrhaging and other complications (e.g., acute heart failure, cardiac arrhythmia, brain infarction and gastric ulcer)
Nattenmüller et al.^51^	2019	RS	1	Germany	296	‐	63.7 ± 11.9^a^	210 (71)	26 ± 4.2 [16–40]^a^	All rectal	‐	180 (61)	During hospital stay	1. Total medical complications 2. Cardiac 3. Pulmonary 4. Sepsis 5. UTI 6. Total surgical complications 7. AL 8. Wound infection 9. Bleeding 10. Abscesses 11. Bladder dysfunction 12. Burst abdomen	‐	Medical: Cardiac, pulmonary, sepsis and UTI Surgical: AL, wound infection, bleeding, abscesses, bladder dysfunction and burst abdomen
Okugawa et al.^52^	2018	RS	1	Japan	308	1. Total myopenia (155) 2. Myosteatosis (153)	>67 (67 is median) 159 1. 71 2. 58	183 (62)	‐	Right colon, 93 Left colon, 215	‐	‐	30 days after surgery	Infectious complications	‐	Infectious complications: Wound infection (superficial or deep infection that required treatment with antibiotic agents or wound drainage), intra‐abdominal abscess (intra‐abdominal fluid collection associated with fever or leucocytosis that discharged spontaneously or required surgical or radiologically guided drainage, with positive blood or fluid culture), respiratory tract infection (respiratory symptoms and signs and infiltrate on chest radiography associated with fever or leucocytosis requiring antibiotic drug treatment), UTI, cholecystitis and *Clostridium difficile*‐associated enteritis. Remote infection included postoperative infectious complication, except for superficial and organ space SSI
Olmez et al.^53^	2021	RS	1	Turkey	160	1. Total 2. SA (73) 3. No‐SA (87)	1. 62.4 ± 12.6^a^ 2. 66.03 ± 10.7^a^ 3. 59.38 ± 13.4^a^	1. 93 (58) 2. 42 (58) 3. 52 (59)	1. 27.8 ± 4.5^a^ 2. 26.9 ± 4.5^a^ 3. 28.5 ± 4.4^a^	All colon, inclusion criterion	1. Laparoscopic, 44 (28) 2. Laparoscopic, 18 (25) 3. Laparoscopic, 26 (30)	1. Chemotherapy, 3 (2) 2. Chemotherapy, 1 (1) 3. Chemotherapy, 2 (2)	10.64 ± 6.67 days	1. Minor complications 2. Major complications 3. Mortality	Clavien–Dindo classification	Minor: CD1–2 Major: CD ≥ 3
Park et al.^54^	2015	RS	1	Korea	543	1. Low VFV (407) 2. High VFV (136)	1. 61.9 ± 10.8^a^ 2. 66.4 ± 8.5^a^	1. 205 (50) 2. 106 (78)	1. <25: 333 (82) ≥25: 78 (58) 2. <25: 57 (42) ≥25: 72 (18)	1. Colon, 252 (62) Rectum, 155 (38) 2. Colon, 105 (77) Rectum, 31 (23)	‐	1. Chemoradiotherapy, 42 (10) 2. Chemoradiotherapy, 9 (7)	30 days after surgery	1. Overall complication 2. Minor complication 3. Major complication	Clavien–Dindo classification	Overall: Any CD Minor: CD1–2 Major: CD ≥ 3
Pedrazzani et al.^55^	2020	RS	1	Italy	261	1. Total 2. VO (173) 3. No‐VO (88) 4. SO (87) 5. No‐SO (174)	1. 67.9 ± 11.7^a^ 2. 69.9 ± 11.0^a^ 3. 64.4 ± 12.3^a^ 4. 73.3 ± 9.3^a^ 5. 65.1 ± 11.8^a^	1. 148 (57) 2. 97 (56) 3. 51 (58) 4. 49 (56) 5. 99 (57)	‐	1. Colon, 181 (72) Rectum, 72 (28) 2. Colon, 126 (73) Rectum, 47 (27) 3. Colon, 53 (71) Rectum, 25 (29) 4. Colon, 67 (77) Rectum, 20 (23) 5. Colon, 122 (70) Rectum, 52 (30)	‐	1. 41 (16) 2. 31 (18) 3. 10 (11) 4.10 (12) 5. 31 (18)	30 days after surgery	1. Overall complication 2. Minor 3. Major 4. General complication 5. Respiratory infectious 6. Cardiac 7. Surgical complications 8. Anastomotic leak 9. SSIs 10. Prolonged postoperative ileus 11. Mortality	Clavien–Dindo classification	Overall: Any CD Minor: CD1–2 Major: CD ≥ 3
Reisinger et al.^3^	2015	PS	1	The Netherlands	310	‐	>70 years: 159 (51)	155 (50)	>25: 182 (59)	Colon, 205 (66) Rectum, 105 (33)	Laparoscopic, 33 (11)	‐	30 days after surgery or within same period of hospital admission	1. AL 2. Mortality 3. Sepsis	‐	AL: Surgically and/or radiologically and/or intra‐abdominal abscess diagnosed by CT examination or treated by percutaneous drainage or surgery Sepsis and septic shock: Defined as ≥2 of the following criteria positive: (1) temperature >38°C or <36°C; (2) heart rate >90 b.p.m.; (3) respiratory rate >20 breaths/min or PaCO_2_ < 32 mmHg; and (4) white blood cell count >12 × 10^9^/L, <4 × 10^9^/L or >10% immature (band) forms plus documented infection and hypotension despite adequate fluid resuscitation (in case of septic shock)
Souwer et al.^56^	2020	RS	1	The Netherlands	174	‐	78 ± 5.1^a^	89 (51)	<25: 79 (45) ≥25: 95 (55)	Colon, 143 (82) Rectum, 31 (18)	Laparoscopic, 114 (66) Open, 60 (34)	Chemoradiotherapy, 9 (5)	In‐hospital and out‐of‐hospital morbidity within 30 days of surgery	1. Any complication 2. Any surgical complication 3. Need for reintervention 4. AL 5. Pulmonary complication 6. Cardiac complication 7. Severe complication 8. Mortality	‐	Surgical complications: Wound infections, ileus and complications that needed (surgical) intervention (including anastomotic leaks) Cardiopulmonary complications: Pulmonary complications (pneumonia, atelectasis, pulmonary embolism, pulmonary insufficiency or other pulmonary complications) and cardiac complications (myocardial infarction, heart failure, arrhythmia, angina pectoris, cardiac arrest or other cardiac complications) Severe complications were defined as complications leading to an ICU admission (more than 2 days), the need for a (surgical) reintervention, a prolonged hospital stay (more than 14 days) or postoperative mortality
Springer et al.^57^	2022	RS	1	The United States	85	1. Total 2. No‐SA (56) 3. SA (29)	1. 59 (51–66)^b^ 2. 59 (51–66)^b^ 3. 59 (55–66)^b^	1. 52 (63) 2. 34 (63) 3. 18 (62)	1. 28 (24–32)^b^ 2. 29 (24–33)^b^ 3. 27 (24–30)^b^	All rectal, inclusion criterion	Laparoscopic + transanal, 85 (100)	1. 69 (85) 2. 45 (87) 3. 24 (83)	Within 30 days	1. Any complications 2. Ileus 3. Urinary retention 4. SSI 5. SBO 6. UTI 7. Anastomotic leak 8. Pelvic abscess 9. Renal failure	‐	‐
Tamagawa et al.^58^	2018	RS	1	Japan	82	1. Total 2. SA (40) 3. No‐SA (42)	1. 86.1 ± 4.9^a^ 2. 86.4 ± 4.8^a^ 3. 85.6 ± 5.0^a^	1. 38 (46) 2. 19 (48) 3. 19 (45)	1. 20.7 ± 3.9^a^ 2. 21.7 ± 4.2^a^ 3. 19.8 ± 3.1^a^	1. Colon, 72 Rectum, 9 2. Colon, 35 (87) Rectum, 5 (13) 3. Colon, 18 (43) Rectum, 24 (57)	‐	‐	30 days after surgery or during hospitalization	Surgical complication	Clavien–Dindo classification	Surgical: CD ≥ 2 TIA, SSI, ileus, pneumonia, colitis, UTI, renal failure, pleural effusion, intraperitoneal abscess, AL, gallbladder perforation and heart failure
Tankel et al.^69^	2020	RS	1	Israel	185	1. Total 2. TPI SA (46) 3. TPI no‐SA (139) 4. HUAC SA (44) 5. HUAC no‐SA (141)	1. >75: 59 (41) 2. >75: 18 (10) 3. >75: 41 (22) 4. >75: 22 (12) 5. >75: 37 (20)	1. 91 (49) 2. 23 (12) 3. 68 (37) 4. 23 (12) 5. 68 (37)	‐	All right hemicolon	‐	‐	Within 30 days after surgery	1. Abdominal complications 2. Respiratory complications 3. Renal complications 4. Cardiac complications 5. Wound complications 6. Serious complications 7. Total complications	Clavien–Dindo classification	Abdominal: Ileus, SBO and intra‐abdominal bleeding Respiratory: Atelectasis, pulmonary oedema, pulmonary embolus, pneumonia and respiratory failure Renal: Urinary retention, acute kidney injury and UTI Cardiac: Arrhythmia and myocardial infarction Wound: Bleeding and infection Serious: CD ≥ 3
Uehara et al.^60^	2022	RS	1	Japan	262	1. SA (49) 2. No‐SA (213)	1. 67 [38–86]^b^ 2. 63 [28–84]^b^	1. 45 (92) 2. 144 (68)	1. 20.4 [16.0–24.9]^b^ 2. 22.7 [15.5–35.0]^b^	All rectum, inclusion criterion	1. Minimally invasive, 41 (84) Open, 8 (16) 2. Minimally invasive, 186 (87) Open, 27 (13)	‐	Within 30 days after surgery	1. Overall complications 2. CD ≥ 3 complications 3. Postoperative infections 4. SSI 5. AL 6. Wound infection 7. Intra‐abdominal abscess 8. Remote infection 9. UTI 10. Pneumonia 11. Enteritis 12. Bowel obstruction 13. Chyle leak 14. Urination disorder 15. Anastomotic bleeding	Clavien–Dindo classification	Postoperative infections: SSIs and remote infections (pneumonia, UTI, catheter‐associated bloodstream infections and antibiotic‐associated enteritis)
van der Kroft et al.^61^	2018	PS	1	The Netherlands	63	1. Total 2. No‐SA (30) 3. SA (33)	1. 69 ± 10.5^a^ 2. <70: 18 (55) ≥70: 12 (40) 3. <70: 15 (45) ≥70: 18 (60)	2. 19 (49) 3. 20 (51)	1. 26^a^ 2. <30: 28 (47) ≥30: 2 (67) 3. <30: 32 (53) ≥30: 1 (33)	1. Colon, 46 (73) Rectum, 17 (17) 2. Colon, 20 (43) Rectum, 10 (56) 3. Colon, 26 (57) Rectum, 7 (44)	1. Laparoscopic, 37 (56) Open, 26 (44) 2. Laparoscopic, 18 (49) Open, 12 (46) 3. Laparoscopic, 19 (51) Open, 14 (54)	‐	Within 30 days after surgery	Complications CD ≥ 2	Clavien–Dindo classification	‐
van Vugt et al.^62^	2018	PS	7	The Netherlands	816	1. High skeletal muscle mass (404) 2. Low skeletal muscle mass (412) 3. High skeletal muscle density 4. Low skeletal muscle density	1. 66 (59–74)^b^ 2. 73 (65–79)^b^ 3. 63 (56–69)^b^ 4. 74 (66–80)^b^	1. 255 (63) 2. 185 (45) 3. 189 (66) 4. 249 (48)	1. <20: 2 (1) 20–24.9: 171 (42) ≥25.0: 231 (57) 2. <20: 25 (6) 20–24.9: 152 (37) ≥25.0: 235 (57) 3. <20: 9 (3) 20–24.9: 97 (34) ≥25.0: 182 (63) 4. <20: 18 (3) 20–24.9: 224 (43) ≥25.0: 282 (54)	1. Colon, 273 (68) Rectum, 131 (32) 2. Colon, 307 (75) Rectum, 105 (25) 3. Colon, 190 (66) Rectum, 98 (34) 4. Colon, 387 (74) Rectum, 137 (26)	1. Laparoscopic, 197 (49) Open, 161 (40) Conversion, 44 (11) 2. Laparoscopic, 198 (48) Open, 180 (44) Conversion, 33 (8) 3. Laparoscopic, 153 (53) Open, 107 (37) Conversion, 26 (9) 4. Laparoscopic, 241 (46) Open, 232 (44) Conversion, 50 (10)	1. 111 (28) 2. 97 (24) 3. 92 (32) 4. 115 (22)	Within 30 days after surgery, during hospital admission or during readmission within 30 days after discharge	1. Overall complications 2. Severe complications	Clavien–Dindo classification	Severe: CD ≥ 3a
Verduin et al.^63^	2021	RS	8	The Netherlands	2370	1. AL 2. No‐AL	70.8 (10.6)	1193 (50.3)	25.7 (4.0)	All colon	‐	‐	Within 30 days after surgery	AL	‐	AL: AL requiring surgical reintervention within the first 30 days
Watanabe et al.^64^	2014	PS	1	Japan	338	1. VFA < 100 (194) 2. VFA ≥ 100 (144)	1. 64.8 [35–87]^a^ 2. 66.2 [35–90]^a^	1. 72 (37) 2. 107 (74)	1. <25: 169 (87) ≥25: 25 (13) 2. <25: 78 (54) ≥25: 66 (46)	All colon	‐	‐	Within 30 days after surgery	1. Total 2. AL 3. SSI 4. SBO	‐	‐
Yang et al.^65^	2019	RS	1	China	417	1. Total	1. 57.9 ± 11.3^a^	1. 251 (60)	1. 23.3 ± 3.3^a^	1. Colon, 220 (53) Rectum, 197 (47)	‐	‐	Within 30 days after surgery or before hospital discharge	Overall postoperative complications	Clavien–Dindo classification	‐
Zhai et al.^66^	2019	RS	1	China	107	1. BMI obese (15) 2. BMI no‐obese (92) 3. V/S obese (95) 4. V/S no‐obese (12) 5. VFA obese (73) 6. VFA no‐obese (34)	1. 68.4 ± 11.1^a^ 2. 67.5 ± 7.3^a^ 3. 68.6 ± 10.6^a^ 4. 67.2 ± 15.3^a^ 5. 69.6 ± 9.1^a^ 6. 65.8 ± 14.5^a^	64 (60)	‐	All colon	Open (42) Laparoscopic (65)	‐	Postoperative 30‐day follow‐up	1. Overall complications 2. CD grade complications 3. Severe complications	Clavien–Dindo classification	Overall: CD1–4 Minor: CD1–2 Major: CD3–4
Zhou et al.^67^	2020	RS	1	China	381	‐	65 (16)	234 (61.4)	22.54 (3.27)	Lower, 1/3: 167 (43.8) Middle, 1/3: 131 (33.4) Upper, 1/3: 83 (21.8)	Laparoscopic, 49 (12.9)	‐	During hospital stay or within 30 days after surgery	Overall complications	Clavien–Dindo classification	Overall: CD ≥ 2

Abbreviations: AL, anastomotic leakage; BMI, body mass index; CT, computed tomography; EPSBO, early postoperative small bowel obstruction; HUAC, Hounsfield unit average calculation; ICU, intensive care unit; IQR, inter‐quartile range; MPM, morphologic change of the psoas muscle; no‐AL, no anastomotic leakage; no‐SA, no sarcopenia; no‐SO, no sarcopenic obesity; no‐SSI, no surgical site infection; no‐VO, no visceral obesity; PRF, perirenal fat; POD, postoperative days; PS, prospective study; RS, retrospective study; SA, sarcopenia; SAT, subcutaneous adipose tissue; SBO, small bowel obstruction; SO, sarcopenic obesity; SSI, surgical site infection; TIA, transient ischaemic attack; TPI, total psoas index; UTI, urinary tract infection; V/S, visceral fat‐to‐subcutaneous fat ratio; VFA, visceral fat area; VFV, visceral fat volume; VO, visceral obesity; WHO, World Health Organization.

^a^ Data are presented as mean ± SD/[range].

^b^ Data are presented as median (IQR)/[range].

### Data extraction and risk of bias assessment

The full text of the selected articles was obtained for further review. Data from the included studies were independently extracted by two authors (CPMvH and JGAvW). Collected data included baseline patient and study characteristics (author, year of publication, country, study design, number of centres, number of patients, sex, age, tumour location, type of surgery, inclusion criteria, exclusion criteria and body mass index [BMI]), BC and CT characteristics (time between CT and surgery, level of CT assessment, BC parameters/indexes, contrast‐enhanced CT, used cut‐off values for BC parameters/indexes, BC definitions, Hounsfield units [HU] cut‐off values, number of investigators and if blinded for outcome) and postoperative outcome definitions (complication definitions used, time of follow‐up and severity measurement) and analysis/outcome characteristics (dichotomous/continuous analysis and reported effect measures). The methodological quality of the full‐text papers was evaluated using the Quality in Prognosis Studies (QUIPS) tool.^68^ If no consensus was reached, a third reviewer (JPMD) was consulted. Of the original six domains in the QUIPS tool, four domains were assessed, namely, study participation, prognostic factor measurement, outcome measurement, and statistical analysis and reporting. Study attrition was not assessed because of the study design of the included studies, and study confounding was not assessed because confounding is not a problem in predictive research and therefore not relevant (see *Tables* *S2* *A* and *S2* *B* for the specific questions answered to assess the risk of bias [RoB]). If ≥1 of the four domains was scored high RoB, overall RoB was scored high. If ≥3 domains were scored moderate and the others low, overall RoB was scored moderate. And if ≤2 were scored moderate and the others low, the overall risk was scored low. The overall risk had no consequences for further use of the studies for meta‐analyses, as the aim was to create a broad evaluation of the evidence in both the complete review and potential meta‐analysis.

### Statistical analysis

Statistical analyses were performed with Review Manager 5.4. The retrieved effect measures (odds ratios [ORs] and their 95% confidence interval [CI]) were used for the assessment of the predictive value. If only *P* values were reported in the original article, these were not reported in this SR, as a *P* value of an OR does not say anything about the magnitude of the association, only that the OR is significantly different from 1. Meta‐analysis was performed if ≥3 studies investigated the same BC measurement with the same cut‐off value for the same outcome. ORs from univariable logistic regression analysis were used. ORs from multivariable logistic regression were only synthesized if adjustment for identical confounding variables had been performed. Univariable and multivariable logistic regression results were not combined. If multiple cut‐offs were used, an additional meta‐analysis on univariable logistic regression to investigate the overall effect of these factors on the complication was performed. The random‐effects model was used, as we expected heterogeneity between the included studies based on baseline characteristics and BC measurement. To assess statistical heterogeneity, Higgins's *I* ^2^ test was used.^69^ *I* ^2^ < 25% was considered low, *I* ^2^ 25–50% as moderate and *I* ^2^ > 50% as high.

## Results

### Search results

**Figure 1:**
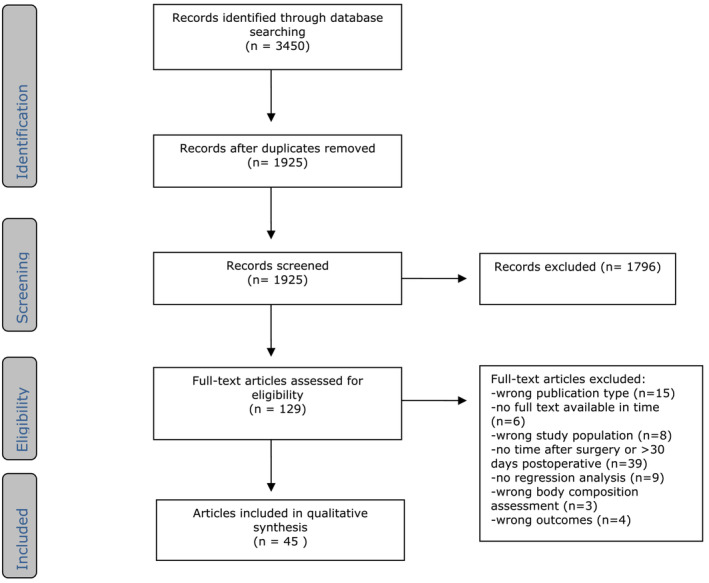
Flow chart of the search and selection procedure of studies.

The literature search generated 3450 references: 996 in PubMed, 1293 in Embase and 1161 in Web of Science. After removing duplicates, 1925 references remained. These references were screened by title and abstract, after which 1796 references were excluded. Of the remaining 129 references, the full text was reviewed, of which 84 were excluded and 45 articles were included.^3^, ^24^, ^25^, ^26^, ^27^, ^28^, ^29^, ^30^, ^31^, ^32^, ^33^, ^34^, ^35^, ^36^, ^37^, ^38^, ^39^, ^40^, ^41^, ^42^, ^43^, ^44^, ^45^, ^46^, ^47^, ^48^, ^49^, ^50^, ^51^, ^52^, ^53^, ^54^, ^55^, ^56^, ^57^, ^58^, ^59^, ^60^, ^61^, ^62^, ^63^, ^64^, ^65^, ^66^, ^67^ The flow chart of the search and selection process is presented in *Figure* *1*.

### Study and patient characteristics

Study characteristics are listed in *Table* *1*. All studies were published between 2012 and 1 August 2022. Most studies had a retrospective design.^24^, ^25^, ^26^, ^27^, ^28^, ^29^, ^31^, ^32^, ^33^, ^34^, ^35^, ^36^, ^37^, ^38^, ^39^, ^40^, ^41^, ^42^, ^43^, ^44^, ^45^, ^47^, ^48^, ^50^, ^51^, ^52^, ^53^, ^54^, ^55^, ^56^, ^57^, ^58^, ^59^, ^60^, ^63^, ^65^, ^66^, ^67^ The majority of the studies were performed in Europe (*n* = 18)^3^, ^24^, ^25^, ^27^, ^28^, ^31^, ^33^, ^36^, ^37^, ^42^, ^43^, ^44^, ^51^, ^55^, ^56^, ^61^, ^62^, ^63^ or Asia (*n* = 18).^29^, ^30^, ^32^, ^34^, ^41^, ^47^, ^48^, ^49^, ^50^, ^52^, ^54^, ^58^, ^60^, ^64^, ^65^, ^66^, ^67^ Only three studies were multicentre.^45^, ^62^, ^63^ The 45 studies contained a total of 16 537 patients. The sample size varied between 47 and 2370 included patients. Males accounted for 56.5% (*n* = 9358) of patients. Ten studies only included patients treated for rectal cancer^24^, ^26^, ^36^, ^37^, ^38^, ^47^, ^51^, ^57^, ^60^; 11 studies only included patients treated for colonic cancer^27^, ^28^, ^31^, ^39^, ^41^, ^42^, ^53^, ^59^, ^63^, ^64^, ^66^; and 24 studies included both patients with colon and rectum carcinoma.^3^, ^25^, ^29^, ^30^, ^32^, ^33^, ^34^, ^35^, ^40^, ^43^, ^44^, ^45^, ^46^, ^48^, ^49^, ^50^, ^52^, ^54^, ^55^, ^56^, ^58^, ^61^, ^62^, ^65^

### Computed tomography characteristics

Time between preoperative CT and surgery was reported in 21 studies and ranged from 2 to 180 days.^30^, ^32^, ^34^, ^39^, ^40^, ^42^, ^45^, ^46^, ^47^, ^49^, ^51^, ^53^, ^55^, ^58^, ^59^, ^60^, ^61^, ^62^, ^65^, ^66^, ^67^ Time was not reported or unclear in 24 studies.^3^, ^24^, ^25^, ^26^, ^27^, ^28^, ^29^, ^31^, ^33^, ^35^, ^36^, ^37^, ^38^, ^41^, ^43^, ^44^, ^48^, ^50^, ^52^, ^54^, ^56^, ^57^, ^63^, ^64^ Only three studies described if CT scans were obtained before or after neoadjuvant therapy.^37^, ^57^, ^62^ Contrast‐enhanced CT images were used in nine studies,^27^, ^34^, ^42^, ^43^, ^45^, ^47^, ^56^, ^59^, ^62^ non‐contrast CT images were used in two studies^49^, ^52^ and the other studies did not describe whether contrast was used or not.^3^, ^24^, ^25^, ^26^, ^28^, ^29^, ^30^, ^31^, ^32^, ^33^, ^35^, ^36^, ^37^, ^38^, ^39^, ^40^, ^41^, ^44^, ^46^, ^48^, ^50^, ^51^, ^52^, ^53^, ^54^, ^55^, ^57^, ^60^, ^61^, ^63^, ^64^, ^65^, ^66^, ^67^ Eleven different levels of measurement were used. The most frequently used level was the third lumbar (L3) vertebra in 26 studies.^3^, ^25^, ^27^, ^29^, ^30^, ^32^, ^34^, ^40^, ^42^, ^43^, ^44^, ^45^, ^46^, ^48^, ^50^, ^53^, ^55^, ^56^, ^57^, ^58^, ^59^, ^60^, ^61^, ^62^, ^65^, ^67^ Other used levels were the L3 to fourth lumbar (L4) intervertebral disc (*n* = 5),^36^, ^37^, ^47^, ^51^, ^63^ the umbilicus (*n* = 6),^26^, ^28^, ^33^, ^41^, ^48^, ^49^ the L4 vertebra (*n* = 3)^27^, ^52^, ^64^ and L4 to fifth lumbar intervertebral disc (*n* = 3).^35^, ^39^, ^66^ Most studies measured the surface area of tissue types and corrected for body height; three studies did obtain the volume of tissue types.^24^, ^51^, ^54^ To distinguish different tissue types, HU cut‐off points were used. To identify visceral adipose tissue (VAT), eight studies used a range from −190 to −30 HU,^24^, ^25^, ^35^, ^39^, ^51^, ^55^, ^64^, ^66^ four a range from −140 to −50 HU,^28^, ^36^, ^37^, ^63^ five a range from −150 to −50 HU^30^, ^32^, ^43^, ^45^, ^67^ and one a range from −150 to −30 HU.^48^ For subcutaneous adipose tissue (SAT), five studies used a range from −190 to −30 HU,^35^, ^39^, ^43^, ^51^, ^66^ two a range from −140 to −50 HU^36^, ^63^ and one a range from −150 to −50 HU.^45^ For intermuscular adipose tissue (IMAT), one study used −190 to −30 HU^43^ and one −140 to −50 HU.^63^ Only one study used different HU cut‐off points for SAT, VAT and IMAT.^43^ For muscle tissue, 187 studies used a range from −29 to +150 HU,^27^, ^29^, ^30^, ^34^, ^38^, ^40^, ^42^, ^43^, ^45^, ^46^, ^50^, ^53^, ^55^, ^56^, ^57^, ^61^, ^62^, ^65^ two a range from +5 to +60 HU^36^, ^37^ and three a range from −30 to +110 HU.^3^, ^52^, ^59^ Eight studies did not or inadequately (i.e., ‘standard HU’ in Frostberg et al.) report the used cut‐off values to distinguish tissue types.^27^, ^31^, ^33^, ^41^, ^49^, ^54^, ^58^, ^60^ Measurements were performed by at least two independent investigators in 13 studies.^3^, ^33^, ^36^, ^37^, ^41^, ^42^, ^46^, ^47^, ^49^, ^57^, ^63^, ^65^, ^66^ Investigators were blinded for outcome in 15 studies,^25^, ^27^, ^30^, ^32^, ^33^, ^34^, ^35^, ^36^, ^37^, ^45^, ^55^, ^59^, ^65^, ^66^, ^67^; the other studies did not describe whether investigators were blinded (see *Tables* 2A, 2B, 2C for the CT characteristics).

**Table 2A:** Body composition measurement characteristics fat‐related

Study	Year	Time of CT scan before surgery	Neoadjuvant therapy before or after scan	Type of scan (contrast/no contrast)	Level of measurement	Type of measurement	Cut‐off values to identify tissue type (HU)	Corrected for body height/surface	Cut‐off values/definition	Measurement assessed blinded for outcome (yes/no)	Measurements performed by	Handling analysis (continuous/dichotomous/oid)
Baastrup et al.^24^	2019	‐	NA	‐	From diaphragm to pubic symphysis	VFV	−190 to −30	‐	75th percentile of VFV = >5.36 L (0.14–9.21 L) = VO	‐	‐	Dichotomous
Bachmann et al.^25^	2018	‐	NA	‐	L3	VFA	−190 to −30	‐	VO: Male VFA > 133 cm^2^ and female VFA > 91 cm^2^	Yes	One individual, twice	Continuous
Ballian et al.^26^	2012	‐	Unclear	‐	Umbilicus	1. AC 2. VFA 3. SFA 4. TFA 5. VFA/SFA ratio	‐	‐	‐	‐	‐	Continuous
Cakir et al.^28^	2015	‐	NA	‐	Umbilicus	VFA	−140 to −50	‐	VO: VFA > 100 cm^2^	‐	‐	Dichotomous
Chen et al.^30^	2018	Within 1 month	NA	‐	L3	VFA	−150 to −50	‐	VO: Male VFA > 130 cm^2^ and female VFA > 90 cm^2^	Yes	One researcher, supervised by radiologist	Dichotomous
der Hagopian et al.^31^	2018	‐	NA	‐	Left renal vein following the anterior renal fascia to the lateroconal ligament encircling the posterior perirenal fat including the retroperitoneal fat posterior to the posterior layer of the renal fascia	PRF	‐	‐	PRF < 40 or ≥40 cm^2^	‐	Single investigator	Dichotomous
Dong et al.^32^	2022	Within 1 month	NA	‐	L3	VFA	−150 to −50	‐	VO: VFA > 130 cm^2^ in men and VFA > 90 cm^2^ in women	Yes	One radiologist	Dichotomous
Frostberg et al.^33^	2021	‐	NA	‐	Umbilicus and S1	1. VFA 2. SAT	Standard HU	‐	1. VO: VFA > 130 cm^2^ 2. Low: <25th percentile Moderate: 25th–75th percentile High: >75th percentile	Yes	Multiple radiologists	Dichotomous and categorical
He et al.^35^	2021	‐	NA	‐	L4–L5 intravertebral space	1. VFA 2. VFA/SFA 3. SFA	−190 to −30	‐	‐	Yes	One radiologist	Continuous
Heus et al.^36^	2019	‐	Unclear	‐	L3–L4 intravertebral disc	1. VFA 2. SFA (TFA–VFA)	−140 to −50	‐	VO: VFA ≥ 100 cm^2^	Yes	One analyst	Dichotomous
Heus et al.^37^	2016	‐	After	‐	L3–L4 intravertebral disc	VFA	−140 to −50	‐	VO: >100 and >130 cm^2^	Yes	Multiple trained analysts	Dichotomous
Kuritzkes et al.^39^	2018	Within 2 months	NA	‐	L4–L5 intravertebral space	1. VFA 2. SFA 3. TFA 4. VFA/TFA ratio	−190 to −30	‐	‐	‐	Single reviewer	Continuous
Looijaard et al.^43^	2019	‐	NA	Contrast‐enhanced	L3	1. IMAT 2. SAT 3. VAT	1. −190 to −30 2. −190 to −30 3. −150 to −50	‐	No cut‐off values, using *Z* scores IMATI%: IMAT/(IMAT + SM) * 100	‐	One researcher, second in case of doubt	Continuously, using gender‐specific standardized *Z* scores
Martin et al.^45^	2018	Average time 30 days	NA	‐	L3	VAT SAT	−150 to −50	VATI (cm^2^/m^2^)	‐	Yes	One observer	Continuously, using gender‐specific standardized *Z* scores
Morimoto et al.^48^	2019	‐	Unclear	‐	Umbilicus	VFA	−150 to −30	‐	VO: VFA ≥ 100 cm^2^	‐	‐	Dichotomous
Nakanishi et al.^50^	2022	Approximately 3 months	NA	Non‐contrast	Umbilicus	1. VFA 2. SFA 3. VFA/SFA ratio	Automated	‐	VFA: ≥100 or <100 cm^2^ SFA: ≥100 or <100 cm^2^ VFA/SFA ratio: ≥0.70 or <0.70	‐	Two clinicians	Dichotomous
Nattenmüller et al.^51^	2019	Mean 27.99 (SD 31.7) days	NA	‐	1. L3/L4 2. Th11/12 to L5/S1 3. S1 to upper edge symphysis	1. TAT VAT SAT VAT/SAT 2. TATV VATV SATV 3. TATV VATV SATV	−190 to −30	‐	‐	‐	‐	Continuous
Park et al.^52^	2015	‐	Unclear	‐	1. S1 to 12.5 cm above	VFV	Automated	‐	Low VFV: VFV < 1.92 dm^3^ High VFV: VFV > 1.92 dm^3^	‐	‐	Dichotomous
Pedrazzani et al.^55^	2020	Within 30 days	Unclear	‐	L3	VAT	−190 to −30	‐	VO: Males VAT > 163.8 cm^2^ and females VAT > 80.1 cm^2^ No‐VO: Males VAT < 163.8 cm^2^ and females VAT < 80.1 cm^2^	Yes	One experienced radiologist	Dichotomous
Verduin et al.^63^	2021	‐	NA	‐	L3–L4 intravertabral disc	1. VF 2. SF 3. MF	−140 to −50	‐	‐	‐	Two observers	Continuous
Watanabe et al.^64^	2014	‐	NA	‐	L4	VFA	−190 to −30	‐	VO: VFA ≥ 100 cm^2^ No‐VO: VFA < 100 cm^2^	‐	‐	Dichotomous
Zhai et al.^66^	2019	Within 30 days	NA	‐	L4–L5 intravertebral disc	1. VFA 2. SFA 3. VFA/SFA ratio	−190 to −30	‐	1. VO: VFA ≥ 100 cm^2^ 3. VO: V/S ≥ 0.4	Yes	Two trained radiologists	Dichotomous
Zhou et al.^67^	2020	Within 1 month	NA	‐	L3	VFA	−150 to −50	‐	High VFA: VFA > 117.9 cm^2^ for men and VFA > 76.9 cm^2^ for women	Yes	One trained investigator	Dichotomous

Abbreviations: AC, abdominal circumference; CT, computed tomography; HU, Hounsfield units; IMAT, intramuscular adipose tissue; L3, third lumbar vertebra; L4, fourth lumbar vertebra; L5, fifth lumbar vertebra; MF, muscle fat; NA, not applicable; PRF, perirenal fat; S1, first sacral vertebra; SAT, subcutaneous adipose tissue; SATV, subcutaneous adipose tissue volume; SF, subcutaneous fat; SFA, subcutaneous fat area; SM, skeletal muscle; TAT, total adipose tissue; TATV, total adipose tissue volume; TFA, total fat area; Th11/12, 11th/12th thoracic vertebra; VAT, visceral adipose tissue; VATI, visceral adipose tissue index; VATV, visceral adipose tissue volume; VF, visceral fat; VFA, visceral fat area; VFV, visceral fat volume; VO, visceral obesity.

**Table 2B:** Body composition measurement characteristics muscle‐related

Study	Year	Time of CT scan before surgery	Neoadjuvant therapy before or after scan	Type of scan (contrast/no contrast)	Level of measurement	Type of measurement	Cut‐off values to identify tissue type (HU)	Corrected for body height/surface	Cut‐off values/definition	Measurement assessed blinded for outcome (yes/no)	Measurements performed by	Handling analysis (continuous/dichotomous/other)
Boer et al.^27^	2016	‐	NA	Venous phase	L3 L4 superior L4 inferior	1. TAMA 2. TPA 3. HU TAMA	−29 to +150	1. TPI (cm^2^/m^2^) 2. TAMI (cm^2^/m^2^)	1. SA: Sex‐specific cut‐off value below the median	Yes	One individual	Continuous and dichotomous
Chai et al.^29^	2021	‐	NA	‐	L3	SMA	−29 to +150	SMI (cm^2^/m^2^)	SA: Males: SMI < 43 cm^2^/m^2^ if BMI < 25 kg/m^2^ or 53 cm^2^/m^2^ if BMI ≥ 25 kg/m^2^ Females: SMI < 38.5 cm^2^/m^2^	‐	‐	Dichotomous
Hanaoka et al.^34^	2017	Within 30 days	NA	Venous phase	L3	1. MPM 2. TPA	−29 to +150	2. TPI (mm^2^/m^2^)	MPM score: Mean of the respective ratios of the short‐axis to long‐axis ratios of the right and left psoas muscles Grade 0: >2/3 Grade 1: 1/2 < − ≤ 2/3, grade 2: 1/3 < − ≤ 1/2, grade 3: 1/4 < − ≤ 1/3, grade 4: ≤1/4	Yes	One investigator	Dichotomous/categorical and continuous
Heus et al.^36^	2019	‐	Unclear	‐	L3–L4 intravertebral disc	SMA	+5 to +60	‐	‐	Yes	One analyst	Dichotomous
Jochum et al.^38^	2019	Unclear, used CT scan is initial staging scan (CT scan performed within 30 days of initial diagnosis)	Unclear	‐	L3	SMA	−29 to +150	SMI (cm^2^/m^2^)	SA: Male SMI < 52.4 cm^2^/m^2^ and female SMI < 38.5 cm/m^2^	‐	‐	Dichotomous
Lieffers et al.^40^	2012	On average 31 days	NA	‐	L3	SMA	−29 to +150	SMI (cm^2^/m^2^)	SA: Female SMI < 38.5 cm^2^/m^2^ and male SMI < 52.4 cm^2^/m^2^	‐	‐	Dichotomous
Looijaard et al.^42^	2020	Median 31 days (IQR 23.0–42.0 days)	NA	Contrast‐enhanced	L3	1. SMA 2. MD	1. −29 and +150	‐	SM%: Dividing SMA of a specific muscle group by total SM surface area multiplied by 100	‐	One researcher, trained and certified	Continuous
Looijaard et al.^43^	2019	‐	NA	Contrast‐enhanced	L3	SMA	1. −29 to +150	SMI (cm^2^/m^2^)	No cut‐off values, using *Z* scores	‐	One researcher, second in case of doubt	Continuously, using gender‐specific standardized *Z* scores
Margadant et al.^44^	216	‐	NA	‐	L3	MD psoas	NA	Hounsfield unit average calculation	Low MD: ≤22.0 HU/mm^2^ for males and ≤23.5 HU/mm^2^ for females (<25th percentile)	‐	One researcher	Continuous and dichotomous
Martin et al.^45^	2018	Average time 30 days	NA	‐	L3	1. SMA 2. MD	−29 to +150	1. SMI (cm^2^/m^2^)	‐	Yes	One observer	Continuously, using gender‐specific standardized *Z* scores
Maurício et al.^46^	2018	Within 60 days	Unclear	‐	L3	SMA	−29 to +150	SMI (cm^2^/m^2^)	Low SMI: For women, SMI < 41 cm^2^/m^2^; for men, SMI < 53 cm^2^/m^2^ if BMI ≥ 25 kg/m^2^ and SMI < 43 cm^2^/m^2^ if BMI < 25 kg/m	‐	One researcher	Dichotomous
Mizuuchi et al.^47^	2022	Within 2 months	NA	Contrast	L3–L4 intervertebral disc	PMA	‐	PMI (cm^2^/m^2^)	PMI > 812.67 cm^2^/m^2^	‐	Two observers	Dichotomous
Nakanishi et al.^50^	2018	‐	NA	‐	L3	1. SMA	−29 to +150	1. SMI (cm^2^/m^2^)	SA: Female SMI < 38.5 cm^2^/m^2^ and male SMI < 52.4 cm^2^/m^2^	‐	‐	Dichotomous
Okugawa et al.^52^	2018	‐	NA	No contrast	L4	1. PMA 2. IMAC	1. −30 to +110	1. PMI (cm^2^/m^2^)	1. Myopenia: Male PMI < 286.8 and female PMI < 210.6 2. Myosteatosis: Male IMAC > −0.36 and female IMAC > −0.2	‐	‐	Dichotomous
Olmez et al.^53^	2021	Within 1 month	Unclear	‐	L3	TPA	−29 to +150	PMI (mm^2^/m^2^)	SA: Male PMI < 545 mm^2^/m^2^ and female PMI < 385 mm^2^/m^2^	‐	‐	Dichotomous
Reisinger et al.^3^	2015	‐	NA	‐	L3	SMA	−30 to +110	SMI (cm^2^/m^2^)	SA: Male SMI < 52.4 cm^2^/m^2^ and female SMI < 38.5 cm^2^/m^2^	‐	Two investigators, 10% control by third investigator	Dichotomous
Souwer et al.^56^	2020	‐	Unclear	Contrast‐enhanced	L3	1. SMA 2. MD	−29 to +150	SMI (cm^2^/m^2^)	1. Low SMI: Women SMI < 41 cm^2^/m^2^; men SMI < 53 cm^2^/m^2^ if BMI ≥ 25 kg/m^2^ and SMI < 43 cm^2^/m^2^ if BMI < 25 kg/m^2^ Low SMI: Q1 men: <39.84 cm^2^/m^2^; women: <32.68 cm^2^/m^2^ High SMI: Q4 men: >48.86 cm^2^/m^2^; women: >37.61 cm^2^/m^2^ 2. Low MD: For BMI < 25 kg/m^2^ HU < 41 and BMI ≥ 25 kg/m^2^ HU < 33 Low MD: Q1 men: <23.21 HU; women: <20.08 HU. High MD: Q4 men: >33.18 HU; women: >31.22 HU	‐	One trained investigator	Categorical
Springer et al.^57^	2022	Time of rectal cancer diagnosis	Before	‐	L3	TPA	−29 to +150	PMI (cm^2^/m^2^)	SA: Males PMI ≤ 5.37 cm^2^/m^2^ and females PMI ≤ 4.11 cm^2^/m^2^	‐	Three researchers	Dichotomous
Tamagawa et al.^58^	2018	Within 1 month	NA	‐	L3	TPA	‐	PMI (cm^2^/m^2^)	SA: Male PMI < 11.9 cm^2^/m^2^ and female PMI < 9.6 cm^2^/m^2^	‐	‐	Dichotomous
Tankel et al.^59^	2020	Less than 30 days	NA	Contrast‐enhanced	L3	1. TPA 2. Average HU of psoas (MD psoas)	1. −30 to +110	1. TPI (cm^2^/m^2^) 2. Hounsfield unit average calculation (HUAC)	1. SA (TPI): TPI < 112.1 cm^2^/m^2^ for females and TPI < 160.6 cm^2^/m^2^ for males 2. SA (HUAC): HU < 44.9 for females and HU < 44.5 males	Yes	One trained radiologist	Dichotomous
Uehara et al.^60^	2022	Within 60 days	NA	‐	L3	TPA	‐	PMI (cm^2^/m^2^)	SA: Males PMI < 6.36 cm^2^/m^2^ and females PMI < 3.92 cm^2^/m^2^	‐	‐	Dichotomous
van der Kroft et al.^61^	2018	Within 2 months	NA	‐	L3	1. SMA 2. MD	−29 to +150	SMI (cm^2^/m^2^)	1. SA: SMI < 43 cm^2^/m^2^ for males with BMI < 25.0 kg/m^2^ and <53 cm^2^/m^2^ for males with BMI ≥ 25.0 kg/m^2^ and 41 cm^2^/m^2^ for females 2. Low MD: <34.1 HU High MD: ≥34.1 HU	‐	‐	Dichotomous
van Vugt et al.^62^	2018	Median 33 (IQR 22–47) days	After	Contrast‐enhanced	L3	1. SMA 2. MD	1. –30 to +150	SMI (cm^2^/m^2^)	1. SA: SMI < 41 cm^2^/m^2^ for women and <43 cm^2^/m^2^ for men with BMI < 25 kg/m^2^ and <53 cm^2^/m^2^ for men with BMI ≥ 25 kg/m^2^ 2. Low MD: <41 HU if BMI < 25 kg/m^2^ and <33 HU if BMI ≥ 25 kg/m^2^	‐	‐	Dichotomous
Yang et al.^65^	2019	Within a week, median 2 days [range 1–7 days]	NA	‐	L3	1. SMA	1. −29 to +150	1. SMI (cm^2^/m^2^)	SA: SMI < 52.4 cm^2^/m^2^ for men and SMI < 38.9 cm^2^/m^2^ for women	Yes	Two independent trained observers	Dichotomous

Abbreviations: BMI, body mass index; CT, computed tomography; HU, Hounsfield units; HUAC, Hounsfield unit average calculation; IMAC, intramuscular adipose tissue content; IQR, inter‐quartile range; L3, third lumbar vertebra; L4, fourth lumbar vertebra; MD, muscle density; MPM, morphologic change of the psoas muscle; NA, not applicable; PMA, psoas muscle area; PMI, psoas muscle index; Q1, first quartile; Q4, fourth quartile; SA, sarcopenia; SM, skeletal muscle; SMA, skeletal muscle area; SMI, skeletal muscle index; TAMA, total abdominal muscle area; TAMI, total abdominal muscle index; TPA, total psoas area; TPI, total psoas index.

**Table 2C:** Body composition measurement characteristics combination—Other

Study	Year	Time of CT scan before surgery	Neoadjuvant therapy before or after scan	Type of scan (contrast/no contrast)	Level of measurement	Type of measurement	Cut‐off values to identify tissue type (HU)	Corrected for body height/surface	Cut‐off values/definition	Measurement assessed blinded for outcome (yes/no)	Measurements performed by	Handling analysis (continuous/dichotomous/oid)
Ballian et al.^26^	2012	‐	Unclear	‐	Umbilicus	1. AC	‐	‐	‐	‐	‐	Continuous
Boer et al.^27^	2016	‐	NA	Venous phase	L3 L4 superior L4 inferior	1. TAMA 2. TPA 3. HU TAMA	−29 to +150	1. TPI (cm^2^/m^2^) 2. TAMI (cm^2^/m^2^)	SO: BMI above 25 kg/m^2^ combined with TPA or TAMA below the sex‐specific median	Yes	One individual	Continuous and dichotomous
Chen et al.^30^	2018	Within 1 month	NA	‐	L3	VFA/TAMA ratio	−150 to −50/−29 to +150	‐	VFA/TAMA ratio: Male >1.19 Female >1.74	Yes	One researcher, supervised by radiologist	Dichotomous
Liu et al.^41^	2019	‐	NA	‐	Umbilicus	1. RAT 2. AD 3. RAT × AD	‐	‐	RAT: Largest sagittal distance between the parietal and visceral sides of rectus abdominis AD: Sagittal distance between the bottom of umbilicus and top of vertebra	‐	Three independent operators	Continuous
Looijaard et al.^42^	2019	‐	NA	Contrast‐enhanced	L3	SMA/VAT ratio	−29 to +150/150 to −50	‐	No cut‐off values, using *Z* scores	‐	One researcher, second in case of doubt	Continuously, using gender‐specific standardized *Z* scores
Pedrazzani et al.^55^	2020	Within 30 days	Unclear	‐	L3	1. SMA 2. VAT	1. −29 to +150 2. −190 to −30	‐	SO: VAT/SMA > second tertile No‐SO: VAT/SMA ≤ second tertile	Yes	One experienced radiologist	Dichotomous

Abbreviations: AC, abdominal circumference; AD, abdominal depth; BMI, body mass index; CT, computed tomography; HU, Hounsfield units; L3, third lumbar vertebra; L4, fourth lumbar vertebra; NA, not applicable; RAT, rectus abdominis thickness; SMA, skeletal muscle area; SO, sarcopenic obesity; TAMA, total abdominal muscle area; TAMI, total abdominal muscle index; TPA, total psoas area; TPI, total psoas index; VAT, visceral adipose tissue; VFA, visceral fat area.

### Risk of bias

Only two studies (4.4%) had low RoB in all assessed QUIPS domains; 43 studies (95.6%) failed to have low RoB and hence were considered moderate/high RoB. Looking at the different domains, in study participation, 40 studies had low risk and 5 moderate RoB; in prognostic factor measurement, nine low risk, 15 moderate risk and 21 high RoB; in outcome measurement, 16 low risk, 23 moderate risk and 6 high RoB; and in statistical analysis and reporting, 41 low risk and 4 moderate RoB. Most articles with moderate/high overall RoB did not mention time between CT and surgery and did not give information about who assessed the BC measurements and if the person was blinded for outcomes. Furthermore, there was a lack of (clear) outcome definitions (see *Table* *S2* *B* for which specific points were assessed and the outcomes).

### Body composition measurements

In total, 26 different BC measurements were investigated as predictive factors for short‐term postoperative complications, of which 11 adipose tissue analyses, 8 muscle analyses, 4 combinations of muscle and fat (e.g., sarcopenic obesity [SO]), and 3 other types of measurements. More than 50 different defined complications were used. The different BC measurements assessed are listed below, along with the level of association/predictive value; if there was no association (NA), the value can be found in *Table* *S3*. The included variables in different multivariable models can be found in *Table* *S4*.

#### Adipose tissue measurements

##### Intermuscular adipose tissue or muscular fat

IMAT/muscular fat (MF) was assessed in three studies for associations with two outcomes, namely, severe complications defined as CD classification 3–5^42^, ^43^ and AL.^63^ Looijaard et al. assessed IMAT in univariable and multivariable models for CD3–5 in four muscle groups: rectus, abdominis, lateral muscles psoas muscle and back muscles; only lateral muscles showed an association (univariable OR: 1.051 [1.007–1.097]; multivariable OR: 1.057 [1.011–1.106]).^42^ Looijaard et al. measured IMAT in percentage and in cm^2^ of all skeletal muscles in univariable and multivariable analyses: None of the measurements showed an association with CD3–5 complications.^43^ Verduin et al. assessed MF and AL (univariable NA).^63^

##### Perirenal fat surface

Perirenal fat surface (PRF) was investigated by der Hagopian et al. PRF showed an association with any complications (OR: 2.32 [1.61–3.35]), surgical complications (OR: 2.56 [1.6–4.0]), SSI (OR: 3.03 [1.6–6.5]), wound dehiscence (OR: 11.89 [2.5–55.6]) and infectious complications (OR: 1.91 [1.1–3.2]) in univariable analyses. Intra‐abdominal infection, AL and cardiovascular complications showed NA. Multivariable analyses showed an association with any complication (OR: 1.68 [1.10–2.56]), CD2 (OR: 2.14 [1.24–3.7]) and CD3 (OR: 2.35 [1.01–5.47]) but not with CD1 and CD4–5.^31^

##### Subcutaneous adipose tissue volume

SAT volume (SATV) was analysed on two levels, namely, abdominal and pelvic levels. SATV was assessed for association with wound infection (pelvic OR: 1.138 [1.015–1.275]), burst abdomen (abdominal OR: 1.423 [1.089–1.86]; pelvic OR: 1.365 [1.112–1.677]), total medical complications (abdominal OR: 1.183 [1.016–1.377]; pelvic OR: 1.193 [1.057–1.346]) and cardiac complications (pelvic OR: 1.218 [1.032–1.438]) in univariable analyses. In multivariable analyses, SATV was analysed for association with wound infection (pelvic OR: 1.015 [1.002–1.029]), burst abdomen (abdominal OR: 1.05 [1.016–1.087]; pelvic OR: 1.04 [1.014–1.067]), medical complications (abdominal OR: 1.021 [1.003–1.04]; pelvic OR: 1.019 [1.005–1.034]) and cardiac complications (pelvic OR: 1.024 [1.004–1.045]).^51^

##### Subcutaneous fat thickness

Subcutaneous fat thickness (SFT) was analysed in univariable analysis in two studies and showed NA with any complication^33^ and SSI.^41^

##### Subcutaneous adipose tissue or subcutaneous fat area

Six studies investigated SAT as a predictor for 14 different postoperative outcomes.^26^, ^36^, ^39^, ^43^, ^49^, ^63^ An association was found in univariable analyses with pneumonia (*P* = 0.031), overall complications (*P* = 0.003) and wound infection (*P* = 0.048)^36^ and not with graded complications,^26^ ileus, UTI, CD1, CD2, CD3, CD4,^36^ overall CD3–5,^39^ surgical‐related complications CD3–5^43^ and early postoperative small bowel obstruction (EPSBO).^49^ AL did not show an association with SAT in two studies.^36^, ^63^ Overall complications,^36^ complications graded CD3–5^39^ and surgical‐related complications graded CD3–5^43^ showed NA in multivariable analyses.

##### Total fat area or total adipose tissue

Three studies analysed total fat area (TFA) as predictive factor for four outcomes.^26^, ^39^, ^51^ Ballian et al. showed NA with complications graded by Mazeh in univariable analyses.^26^

Kuritzkes et al. investigated TFA and CD3–5 (univariable analysis [*P* = 0.04] and multivariable [NA]).^39^ Total adipose tissue (TAT) was analysed in univariable and multivariable analyses for association with burst abdomen (univariable OR: 1.135 [1.01–1.275]; multivariable OR: 1.013 [1.001–1.025]) and cardiac complications (univariable OR: 1.116 [1.01–1.232]; multivariable OR: 1.012 [1.001–1.022]).^51^

##### Total adipose tissue volume

One study investigated abdominal TATV and pelvic TATV; these were significantly associated with burst abdomen in univariable (abdominal OR: 1.321 [1.097–1.591]; pelvic OR: 1.354 [1.12–1.648]) and multivariable analyses (abdominal OR: 1.026 [1.007–1.046]; pelvic OR: 1.034 [1.012–1.056]).

Pelvic TATV was significantly associated with wound infection in univariable analysis (OR: 1.106 [1.002–1.22]) but not in multivariable analysis, same for abdominal TATV and bladder dysfunction (OR: 1.118 [1.00008–1.249]). Both abdominal TATV and pelvic TATV were associated with overall medical complications in univariable (abdominal OR: 1.11 [1.009–1.217]; pelvic OR: 1.157 [1.042–1.286]) and multivariable analyses (abdominal OR: 1.011 [1.0007–1.02]; pelvic OR: 1.014 [1.002–1.026]). This accounted also for abdominal TATV and pelvic TATV and cardiac complications (abdominal: univariable OR: 1.197 [1.038–1.381] and multivariable OR: 1.023 [1.006–1.04]; pelvic: univariable OR: 1.214 [1.044–1.412] and multivariable OR: 1.021 [1.004–1.04]).^51^

##### Visceral fat area‐to‐subcutaneous fat area ratio

Five studies described visceral fat area (VFA)‐to‐subcutaneous fat area (SFA) ratio (VSR) as a predictive factor for seven postoperative outcomes.^26^, ^35^, ^49^, ^51^, ^66^ VSR was analysed by univariable analyses for any complication by Zhai et al. (NA)^66^ and He et al. (*P* = 0.006).^35^ VSR showed NA with for CD3–5, CD1–4^66^ and complications graded by Mazeh et al.,^26^ but did show an association with UTI (OR: 0.804 [0.649–0.997]).^51^ Multivariable analysis was performed for any complications (OR: 6.103 [1.173–31.748]), EPSBO (OR: 2.25 [1.00–5.06])^49^ and UTI (NA).^51^

##### Visceral adipose tissue volume

VAT volume (VATV) was assessed in three studies for eight different outcomes.^24^, ^51^, ^54^ Nattenmüller et al. analysed VATV in two compartments, namely, the pelvis and the abdomen. Pelvic VATV was analysed for association with surgical complications (univariable OR: 0.915 [0.854–0.981]; multivariable OR: 0.998 [0.998–0.999]) and AL (univariable OR: 0.587 [0.369–0.934]; multivariable OR: 0.933 [0.887–0.98]).^51^ Abdominal VATV was analysed for association with bladder dysfunction (univariable OR: 1.196 [1.015–1.409]; multivariable NA), burst abdomen (univariable OR: 1.336 [1.052–1.697]; multivariable NA) and cardiac complications (univariable OR: 1.284 [1.066–1.547]; multivariable OR: 1.02 [1.0001–1.041]).^51^ Park et al. showed an association between high VATV and CD3–5 (*P* = 0.029) and NA with CD1–2 and overall complications in univariable analysis. In multivariable analysis, high VATV was associated with CD3–5 (OR: 3.336 [1.426–7.806]).^54^ Baastrup et al. analysed VATV, measured from diaphragm to pubic symphysis, which showed NA with AL (univariable) and also NA with any complication (univariable and multivariable).^24^

##### Visceral adipose tissue or visceral fat area

VFA was investigated in 20 articles for 41 different outcomes.^24^, ^25^, ^26^, ^28^, ^30^, ^32^, ^33^, ^35^, ^36^, ^37^, ^39^, ^43^, ^45^, ^48^, ^51^, ^55^, ^63^, ^64^, ^66^, ^67^ Eight studies analysed VFA as a continuous variable^25^, ^26^, ^35^, ^39^, ^43^, ^45^, ^51^, ^63^ and 12 studies as a dichotomous variable.^28^, ^30^, ^32^, ^33^, ^36^, ^37^, ^48^, ^49^, ^55^, ^64^, ^66^, ^67^ Dichotomization in high VFA versus low VFA was based on six different cut‐off values.

#### Meta‐analyses

**Figure 2:**
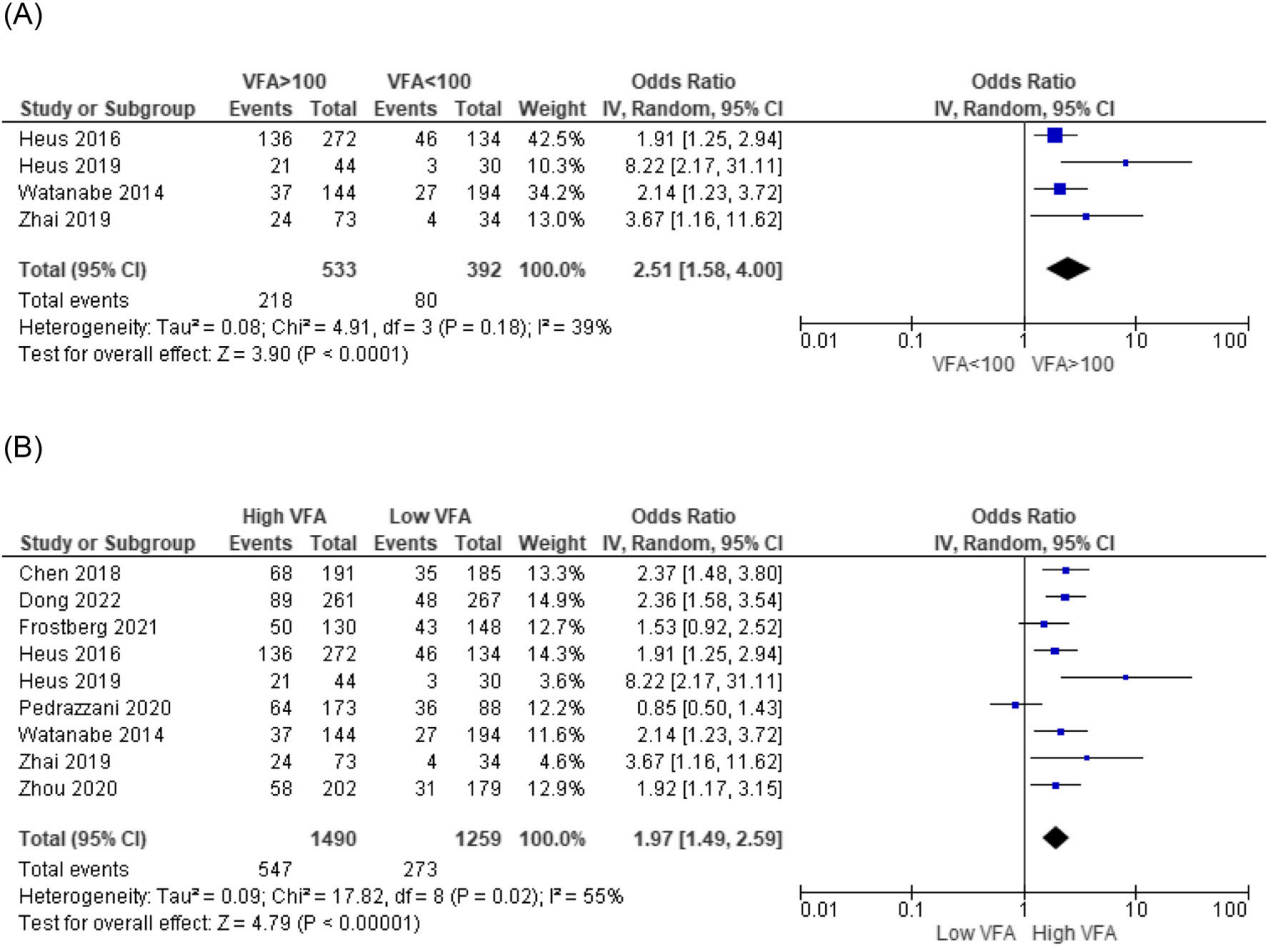
Meta‐analysis of the effect of visceral fat area (VFA) on overall complication using univariable data. (A) Meta‐analysis including only studies that used the cut‐offs of VFA > 100 and VFA < 100. (B) Meta‐analysis including all cut‐off points. CI, confidence interval.

For overall/any complications and AL, it was possible to perform a meta‐analysis of data from univariable analyses. Meta‐analysis of four studies using VFA > 100 cm^2^/VFA < 100 cm^2^ as cut‐off and overall/any complication (three low and one high RoB), including 925 patients, showed that high VFA was significantly associated with development of postoperative complications (OR: 2.52 [1.58–4.00]) with moderate heterogeneity (*I* ^2^ = 39%, *P* = 0.18) (see *Figure* *2* *A*).^36^, ^37^, ^64^, ^66^ Seven other studies investigated VFA and any complications; however, they used VFA as a continuous variable or used different cut‐off points.^25^, ^30^, ^32^, ^33^, ^35^, ^55^, ^67^ Three studies did not show an association.^25^, ^33^, ^55^ The other four studies showed that higher VFA was associated with overall complications (*P* < 0.001),^30^ (*P* < 0.001),^34^ (*P* = 0.016)^35^ and (*P* = 0.009).^67^ An additional meta‐analysis was performed to investigate overall effect of VFA on overall complications regardless used cut‐offs, which showed a significant association (OR: 1.97 [1.49–2.59]) with high heterogeneity (*I* ^2^ = 55%, *P* = 0.02) (see *Figure* *2* *B*).^30^, ^32^, ^33^, ^36^, ^37^, ^55^, ^64^, ^66^, ^67^

**Figure 3:**
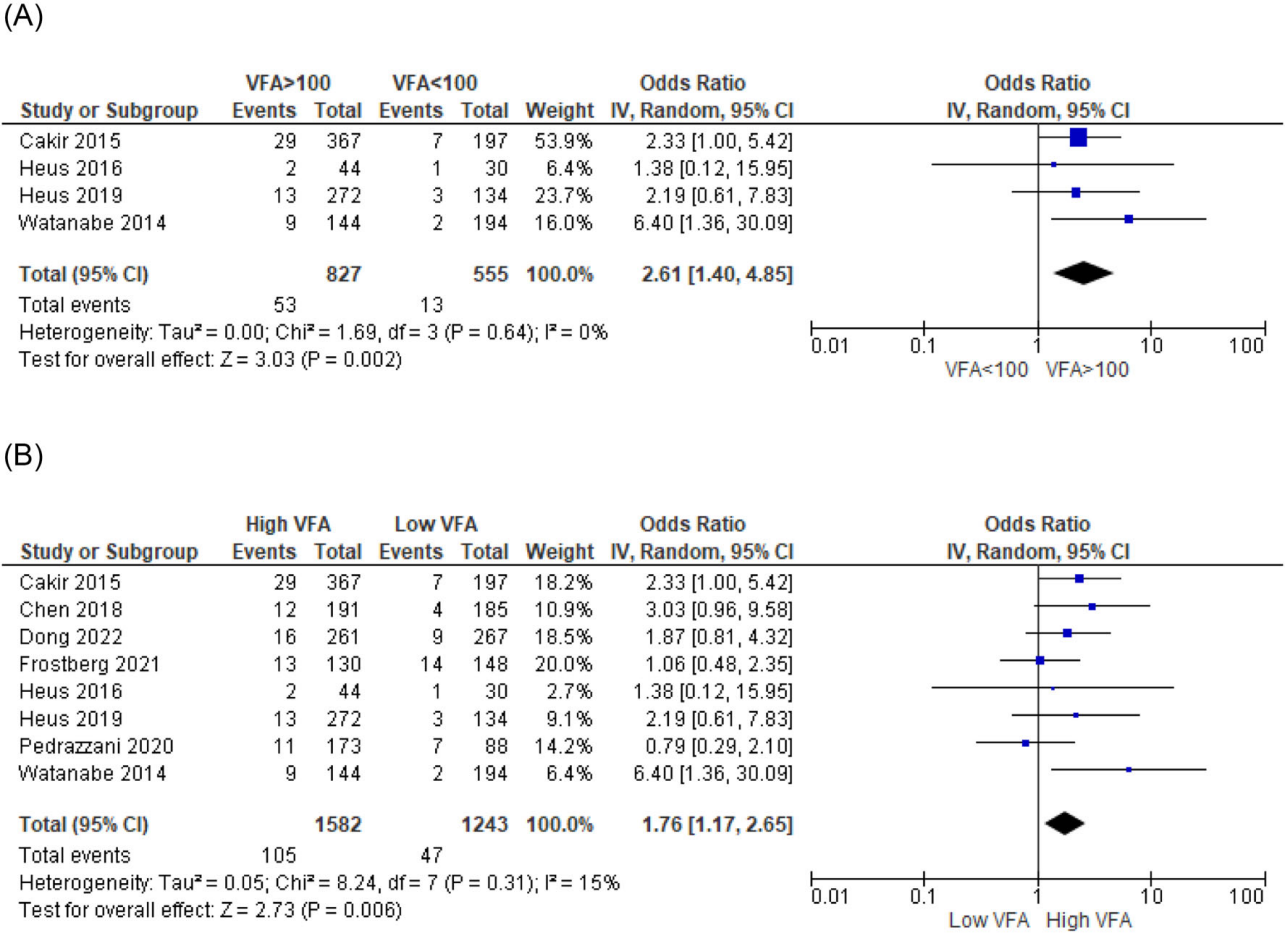
Meta‐analysis of the effect of visceral fat area (VFA) on anastomotic leakage using univariable data. (A) Meta‐analysis including only studies that used the cut‐offs of VFA > 100 and VFA < 100. (B) Meta‐analysis including all cut‐off points. CI, confidence interval.

Four studies using VFA > 100 cm^2^/VFA < 100 cm^2^ as a cut‐off were pooled with AL as an outcome (two low and two high RoB), including 1382 patients. Meta‐analysis showed that high VFA was significantly associated with developing AL (OR: 2.61 [1.40–4.85]) with methodological homogeneity (*I* ^2^ = 0%, *P* = 0.64) (see *Figure* *3* *A*).^28^, ^36^, ^37^, ^64^ Five other studies investigated the association with AL.^32^, ^34^, ^35^, ^57^, ^65^ Only Verduin et al. showed an association (OR: 1.002 [1.001–1.004]).^63^ Additional meta‐analysis using all cut‐offs showed a significant association (OR: 1.76 [1.17–2.65]) with low heterogeneity (*I* ^2^ = 15%, *P* = 0.31) (see *Figure* *3* *B*).^28^, ^30^, ^32^, ^33^, ^36^, ^37^, ^55^, ^64^

Other outcomes that showed an association in univariable analyses were bladder dysfunction (OR: 1.157 [1.009–1.326]), burst abdomen (OR: 1.257 [1.029–1.536]),^51^ CD3–5 (*P* = 0.002),^39^ CD3–5 (OR: 1.341 [1.007–1.787]),^43^ wound infection (*P* = 0.048), ileus (*P* = 0.053), pneumonia (*P* = 0.031),^36^ surgical complications (*P* = 0.001), wound infection (*P* = 0.030), intra‐abdominal abscess (*P* = 0.025), medical complications (*P* = 0.022), pulmonary complications (*P* = 0.023),^32^ ileus (*P* = 0.027),^37^ pneumonia (OR: 2.4 [1.2–4.8]), wound infection (OR: 2.5 [1.1–5.8]),^28^ SSI (*P* = 0.016),^64^ POI (*P* = 0.002)^48^ and respiratory complications (*P* = 0.07).^55^ All analyses that did not show an association (*n* = 33) are displayed in *Table* *S3*.

**Figure 4:**
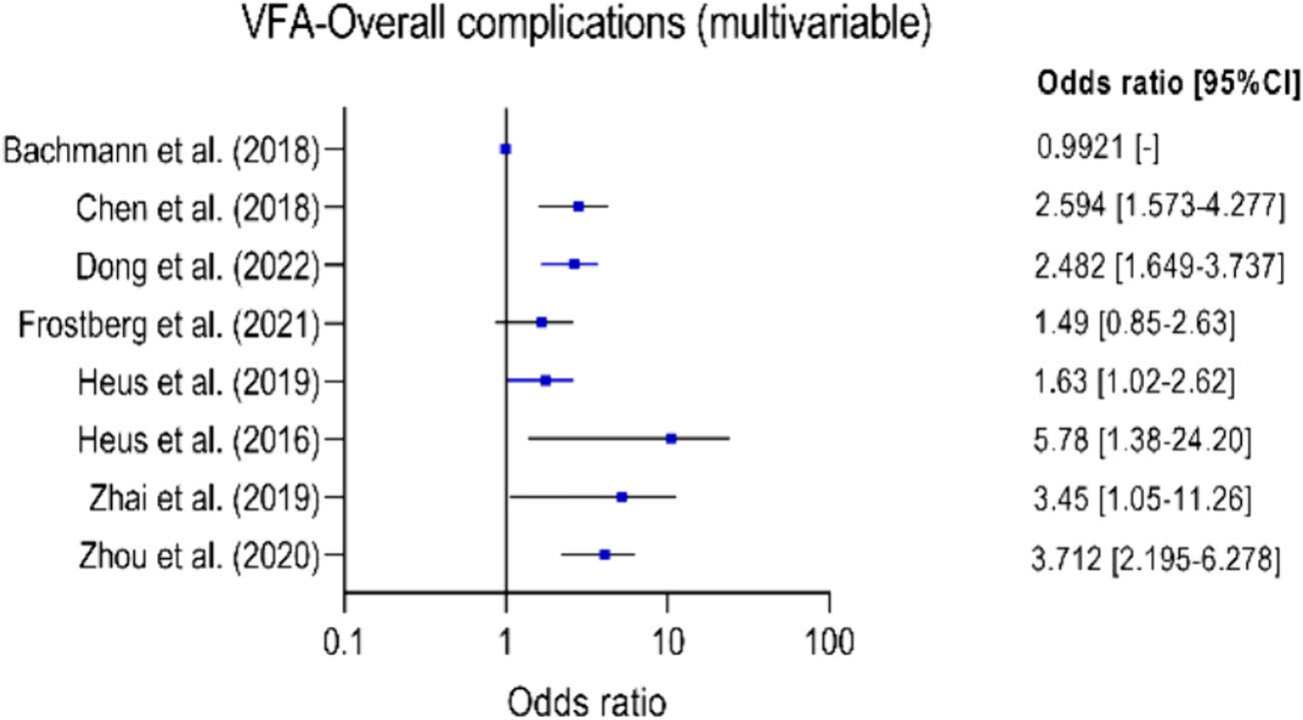
Forest plot of the effect of visceral fat area (VFA) on overall complications from multivariable logistic regression analysis. CI, confidence interval.

VFA was analysed using multivariable logistic regression for CD3–5 in three studies, by Kuritzkes et al. (OR: 2.00 [1.25–3.20]),^39^ by Looijaard et al. (NA)^43^ and by Bachmann et al. (*P* = 0.001).^25^ Overall complications were investigated in eight studies, of which seven showed an association (ORs ranged from 0.9921 [—] to 5.78 [1.38–24.20]; see *Figure* *4*).^25^, ^30^, ^32^, ^33^, ^36^, ^37^, ^66^, ^67^ Due to various variables used in the models, it was not possible to perform a meta‐analysis. Four other outcomes were analysed, namely, surgical complications (OR: 2.060 [1.329–3.191]), medical complications (OR: 2.150 [1.170–3.953]),^32^ POI (OR: 6.2 [1.3–30.4])^48^ and AL (OR: 6.270 [1.325–29.685]).^64^ Cakir et al. analysed pneumonia in multivariable analysis; however, visceral obesity (VO) was not significant (NS), and they did not report the statistics.^28^

##### Visceral fat area‐to‐total fat area ratio

The association between VFA‐to‐TFA ratio and major morbidity was analysed by univariable (*P* = 0.01) and multivariable analyses (OR: 27.90 [0.96–810.08]).^39^

#### Muscle tissue measurements

##### Mean muscle density/radiation attenuation

Muscle density (MD)/radiation attenuation (RA) was assessed in nine studies for 10 different outcomes.^27^, ^42^, ^43^, ^44^, ^45^, ^52^, ^56^, ^61^, ^62^ Five studies dichotomized MD/RA based on four different cut‐off values,^52^, ^56^, ^61^, ^62^ four as continuous variable^27^, ^42^, ^43^, ^45^ and one both dichotomized and continuous.^44^ Boer et al. assessed MD of total abdominal muscle area (TAMA) on three different levels, namely, L3, L4 superior and L4 inferior. Univariable analyses showed an association with any complication at L3, L4 superior and L4 inferior. In multivariable analyses, these associations were still visible: L3 (OR: 0.912 [0.863–0.964]), L4 superior (OR: 0.912 [0.863–0.964]) and L4 inferior (OR: 0.922 [0.875–0.972]). There was NA with severe complications.^27^ Looijaard et al. investigated the association of MD in four different muscle groups (rectus abdominis, lateral muscles, psoas muscle and back muscles) with severe complications. Lateral and psoas muscles were associated in both univariable (OR: 0.952 [0.911–0.995]; OR: 0.946 [0.906–0.987]) and multivariable analyses (OR: 0.952 [0.908–0.998]; OR: 0.944 [0.901–0.989]).^42^ Four studies investigated association between low MD/RA and CD3–5 in univariable analyses of which two showed an association (OR: 1.99 [1.22–3.24] dichotomized; OR: 1.14 [1.06–1.24] continuous)^44^ and *P* = 0.003^62^ and two did not.^43^, ^45^ Three studies performed multivariable analyses and showed an association (OR: 0.684 [0.486–0.962]^43^; OR: 1.80 [1.11–2.97] dichotomized and OR: 1.09 [1.04–1.14] continuous^44^; and OR: 1.87 [1.01–3.46]^62^). Two studies investigated overall/any complications: One study found an association in univariable analysis,^62^ and the other study did not find an association.^56^ AL was investigated by two studies: One showed NA,^56^ and the other did show an association.^44^ Mortality was investigated by two studies, which showed NA.^44^, ^56^ Other investigated outcomes in both univariable and multivariable analyses were infectious complications (univariable OR: 1.74 [1.04–2.91]; multivariable OR: 1.74 [1.02–2.99]),^52^ CD2–5 (univariable *P* = 0.05; multivariable NA),^61^ surgical complications (both NA), pulmonary complication (univariable OR: 0.53 [0.29–0.98]; multivariable NA), cardiac complication (univariable OR: 0.42 [0.20–0.85]; multivariable OR: 0.47 [0.22–0.99]) and severe complications (both NS).^56^

##### Morphologic change of the psoas muscle

Severe morphologic change of psoas muscle (MPM) showed an association with overall complications (OR: 2.93 [1.22–6.99]) and infectious complications (OR: 4.85 [1.63–14.45]) in univariable analysis.^34^

##### Total psoas area

Total psoas area (TPA) was measured at three levels (L3, L4 superior and L4 inferior) and analysed by univariable analysis for overall complications and CD3–5, which showed NA.^27^

##### Psoas muscle index

Psoas muscle index (PMI) was assessed in seven studies for 33 different outcomes.^47^, ^52^, ^53^, ^57^, ^58^, ^59^, ^60^ All studies analysed PMI as a dichotomous variable, with five defined as sarcopenic and non‐sarcopenic^53^, ^57^, ^58^, ^59^, ^60^ and one as myopenic and non‐myopenic.^52^ Mizuuchi et al. investigated the association of PMI with AL CD ≥ 2 (*P* < 0.001) (multivariable analysis showed risk ratio [RR] 3.933 [1.917–8.070]).^47^ An association of sarcopenia measured by PMI was found by univariable analyses for infectious complications (OR: 2.07 [1.23–3.48])^52^; CD3–5 (OR: 3.558 [1.201–10.526])^53^; any complication and small bowel obstruction^57^; surgical complications (OR: 2.726 [1.105–6.728])^58^; respiratory complications, cardiac complications and CD3–5^59^; and any complication (OR: 2.11 [1.12–3.97]), remote infection and chyle leak.^60^ For an overview of all analyses without an association with PMI (*n* = 25), see *Table* *S3*.

Multivariable analyses were performed for infectious complications (OR: 1.96 [1.14–3.37])^52^; CD3–5 (OR: 3.039 [1.008–9.174])^53^; surgical complications (OR: 3.508 [1.154–10.653])^58^; overall complications (OR: 4.44 [1.60–12.38])^57^; and overall complications (NA).^60^

##### Rectus abdominis thickness

Rectus abdominis thickness (RAT) showed an association with SSI in univariable analysis but not in multivariable analysis.^41^

##### Skeletal muscle area

Five studies analysed skeletal muscle area (SMA) and the association with two outcomes.^27^, ^36^, ^42^, ^43^, ^61^ Looijaard et al. analysed in univariable and multivariable analyses the association of SMA of four muscle groups with CD3–5 complications, which did not show an association.^42^

The other studies analysed the total abdominal muscle area (TAMA),^27^, ^37^, ^43^, ^61^ of which one study analysed TAMA on three levels.^27^ Boer et al. performed univariable analyses for the association of TAMA L3, L4 superior and L4 inferior and overall and severe complications; none of the measurements showed an association with the two outcomes.^27^ Furthermore, TAMA was analysed using univariable analysis for the association with overall complications^36^ and CD3–5^61^ and with surgical‐related CD3–5 by univariable and multivariable analyses (two models),^43^ but none showed an association.

##### Skeletal muscle index

Twelve studies investigated the association of skeletal muscle index (SMI) with 13 different postoperative complications.^3^, ^29^, ^38^, ^40^, ^43^, ^45^, ^46^, ^50^, ^56^, ^61^, ^62^, ^65^ Ten studies analysed SMI as a dichotomous variable,^3^, ^29^, ^38^, ^40^, ^46^, ^50^, ^56^, ^61^, ^62^, ^65^ of which seven studies defined as sarcopenic and non‐sarcopenic^3^, ^29^, ^38^, ^40^, ^50^, ^61^, ^65^ and the other three studies as high and low SMI.^46^, ^56^, ^62^ Five different cut‐off values were used to dichotomize SMI. Two studies analysed SMI as a continuous variable.^43^, ^45^ Seven studies analysed overall complications; due to different cut‐off points, pooling of data was not possible.^29^, ^38^, ^43^, ^46^, ^50^, ^62^, ^65^ However, to gain insights in the overall effect, an additional meta‐analysis was performed (see *Figure* *S1A*). In univariable analysis, an association with overall complications was found by Chai et al.,^29^ Jochum et al. (OR: 3.81 [1.13–12.8]),^38^ Maurício et al. (RR 1.89 [1.04–3.42]),^46^ Nakanishi et al.^50^ and Yang et al. (OR: 3.235 [1.719–6.087]).^65^ Two studies did not find an association.^56^, ^62^ Three studies performed multivariable analyses, of which two found an association (OR: 2.96 [1.19–7.35]^29^ and OR: 2.832 [1.346–5.959]^65^) and one did not.^56^ Five studies investigated association with CD3–5 graded complications.^29^, ^43^, ^45^, ^50^, ^62^ Pooling data was not possible because of differences in cut‐off points. To see the overall effect, an additional meta‐analysis was performed (see *Figure* *S1B*). In univariable analyses, an association with CD3–5 was found by Chai et al.,^29^ Nakanishi et al.^50^ and van Vugt et al.^62^ The other studies did not find an association.^43^, ^45^ Four studies performed multivariable analyses, with one study using two models, which did not find an association.^43^ Associations were found by Chai et al. (OR: 2.99 [1.01–8.81]),^29^ Nakanishi et al. (OR: 1.82 [1.13–3.00])^50^ and van Vugt et al. (OR: 1.91 [1.12–3.25]).^62^ AL was analysed by two studies and showed NA.^3^, ^56^ Souwer et al. analysed five other complications in univariable and multivariable analyses, namely, pulmonary complications (both NS), cardiac complications (univariable NA; multivariable OR: 0.44 [0.22–0.90]), severe complications (both NA) and surgical complications (univariable OR: 1.49 [1.04–2.02]; multivariable NA).^56^ Four outcomes were analysed in univariable analysis, of which CD4–5,^50^ CD1 and CD2^65^ showed an association but sepsis did not.^3^ Univariable and multivariable analyses were performed for infectious complications (univariable OR: 4.4 [1.5–13.0]; multivariable OR: 4.6 [1.5–13.9])^40^ and CD2–5 (both NA).^61^

##### Skeletal muscle index combined with muscle density

One study combined SMI with MD as a predictor for CD3–5 complications (univariable NA).^45^

#### Combination of adipose and muscle tissue measurements

##### Sarcopenic obesity

Two studies investigated SO, which is a combination of sarcopenia (measured using TPA, TAMA or SMA) and obesity (measured using BMI or VAT), as a dichotomized variable.^27^, ^55^ Boer et al. analysed SO using two measurements (TPA and TAMA) on three levels (L3, L4 superior and L4 inferior). The univariable analysis of six different SO measurements with any complication showed NA. For severe complications, TPA L3 (OR: 6.4 [1.9–21.3]), TPA L4 superior (OR: 6.4 [1.9–21.3]), TPA L4 inferior (OR: 5.6 [1.7–18.4]) and TAMA L3 (OR: 3.7 [1.2–11.6]) showed an association, but the other two did not.^27^ Pedrazzani et al. investigated SO in univariable analyses for association with mortality, overall complications, CD3–4, general complications, respiratory complications, surgical complications, AL, SSI and infectious complications, NAs were found. Univariable and multivariable analyses were performed for association with cardiac complications (univariable OR: 3.82 [1.45–10.08]; multivariable OR: 3.61 [1.32–9.90]) and prolonged postoperative ileus (univariable OR: 2.37 [1.03–5.45]; multivariable 2.81 [1.18–6.70]).^55^

##### Skeletal muscle area‐to‐visceral fat area ratio

The SMA‐to‐VFA ratio (SVR) was analysed for CD3–5 (univariable and multivariable NA).^43^

##### Visceral fat area‐to‐total abdominal muscle area ratio

The VFA‐to‐TAMA ratio (VTR) showed an association with overall complications in univariable analysis.^30^

##### Combination of visceral fat area/skeletal muscle index/muscle density

One study combined multiple measurements. Combinations investigated were SMI + VFA, VFA + MD and SMI + VO + MD. These did not show an association with CD3–5 complications.^45^

#### Other measurements

##### Abdominal circumference

NA was found between abdominal circumference (AC) and complications graded by Mazeh et al.^26^

##### Abdominal depth

Abdominal depth (AD) showed NA with SSI in univariable analysis.^41^

##### Rectus abdominis thickness × abdominal depth

RAT × AD showed an association with SSI in univariable analysis (OR: 1.007 [1.003–1.010]).^41^

## Discussion

This SR and meta‐analysis gives an overview of the extensive research into the predictive value of different CT scan‐based BC measurements on developing postoperative complications within 30 days after CRC surgery. This study included 45 articles that investigated 26 bc measurements as a predictive factor for a great variety of short‐term postoperative complications. VFA was the most studied AT‐related measurement (*n* = 20), and SMI was the most studied muscle‐related measurement (*n* = 12). Meta‐analysis of univariable analysed data showed that VFA is significantly associated with overall/any complications and AL.

We show that BC measurements on CT scans are frequently performed and investigated as predictors for numerous and diverse postoperative complications, varying from cardiac complications to AL. As most CRC patients will undergo CT scan as part of disease staging, BC measurements are available for a large patient population, and no extra examinations are needed. Thus CT based body composition presents an ideal potential addition to optimize the preoperative risk assessment. The measurements that are currently investigated for postoperative complications can be divided into four categories: (1) fat measurements (IMAT/MF, PRF, SATV, SFT, SAT/SFA, TAT area [TATA]/TFA, TATV, VSR, visceral fat volume [VFV], VFA/VAT and VTR); (2) muscle measurements (MD/RA, MPM, TPA, PMI, RAT, SMA, SMI and SMI + MD); (3) a combination of muscle and fat (SO, SVR, VTR and VFA + SMI + MD); and (4) other measurements (AC, AD and RAT × AD). Most BC measurements are not of predictive value for the investigated outcome, and the reported ORs often range between 1.01 and 2.0 when an association is found. Aggregating results from individual studies might improve statistical power and give more robust effect estimates. However, meta‐analysis was only possible for VFA/VAT and two outcomes due to different cut‐off values for dichotomizing VFA. Meta‐analyses resulted in ORs around 1.5–2, which are relatively low considering the context of the investigated predictive factor. This factor aims to predict an outcome, potentially influencing treatment strategy. An OR of 1.5–2 will probably not change clinical practice, as the risk of the complication is not increased enough.

Besides low ORs, the results described are often ambiguous. Inability to perform meta‐analyses and ambiguous outcomes of studies could be the result of multiple methodological issues that were identified in this SR. These issues can be separated into three categories: CT scan‐related, outcome definition‐related and statistical analysis‐related.

In most studies, it is unclear if contrast‐enhanced or non‐contrast‐enhanced scans were used. Of the 45 included studies, only 9 reported if contrast was used.^42^, ^43^, ^45^, ^47^, ^49^, ^55^, ^56^, ^59^, ^62^ HU values of different tissues are affected by contrast. Because cut‐off values for distinguishing tissue types are based on these HU values, using incorrect cut‐off values could lead to overestimation or underestimation of the amount of muscle and/or AT.^70^ Furthermore, the time between the CT scan and the date of index surgery was not reported or unclear in more than half of the studies. When reported, the time span ranged widely from 7 to 180 days preoperatively. BC is not stable and might change between the date of the scan and index surgery due to dietary interventions, immobilization, neoadjuvant chemotherapy, and/or radiotherapy and illness. Being immobilized or ill, as short as only 4 days, could lead to significant loss of muscle mass.^13^, ^40^, ^71^ If the time span between surgery and scan is too long, BC measurements may not reflect the physical status of the patient at the time of operation and therefore may not be a reliable predictor. The last CT‐related issue is that only 14 studies reported whether investigators were blinded for outcomes.^25^, ^27^, ^30^, ^32^, ^34^, ^35^, ^36^, ^37^, ^45^, ^55^, ^63^, ^65^, ^66^, ^67^ Not blinding investigators could lead to observer and/or detection bias.

Besides CT‐related issues, investigated outcomes were often not clearly described. Most studies only name the complication but lack a definition. Multiple studies graded their complications using CD classification, which gives an indication of the severity of the complication. However, it is unclear what types of complications were scored. This lack of outcome definition could lead to studies indicating they investigate the same outcome but eventually investigate completely different outcomes. For example, the definition of AL, which is a commonly used outcome without a widely accepted definition, will lead to more heterogeneity and hamper the comparability of study results.^10^

The final category comprises statistical analysis‐related issues. BC measurements are continuous variables; however, most studies analyse BC measurements as dichotomous variables. To dichotomize, different cut‐off values are used. Some are based on their own data, using values below or above the median or below or above the 25th/75th percentile; others are based on more generally accepted cut‐off values, whether or not they differentiate between females and males. It is important to take sex differences into account in BC analyses, as it is known, for example, that males with high SFA and VFA, but not females, have an increased risk of infectious complications. This difference might result from increased technical difficulty during the operation or a potential increased systematic inflammatory response.^72^, ^73^ Furthermore, cut‐off values are sometimes obtained from studies investigating other diseases, such as breast cancer, respiratory tract cancer or after pancreatectomy,^15^, ^27^, ^38^, ^40^, ^50^, ^55^, ^59^, ^74^, ^75^ and they do not apply the international guideline for cachexia in cancer.^76^ Or cut‐off values are used that are based on a European–American population for an Asian population or the other way around. As it is known that these populations have different BC, these cut‐off values might not be suitable for other ethnic populations.^77^ Dichotomizing BC measurements may lead to unreliable, non‐generalizable and non‐comparable results.

The goal of most studies was to investigate the predictive value of BC measurements using multivariable logistic regression. Different strategies are used for identifying predictors, including data‐driven, clinically driven or a combination of both. This results in very diverse models. In addition to this diversity, the variables included were sometimes highly questionable, as some variables were part of the outcome, for example, including a variable that inherently relates to the outcome beforehand, such as a specific complication, when the outcome was overall complications, or including variables that can only be determined postoperatively, such as tumour diameter, when the objective is to assess preoperative risk. The combination of very diverse models and questionable variables included in the models impairs the comparability and generalizability of the results.

To overcome the above‐mentioned issues, our recommendation would be to standardize methods for measuring BC on CT scan. This should include reporting which type of scan is used, which HU values are used to differentiate between tissue types, time between scan and operation, and if the investigator was blinded for outcomes or not. Furthermore, outcome definitions should be adequately described, including which complications are included and which definition is used. For statistical analysis, BC measurements should be analysed continuously and after that as a dichotomous variable. For dichotomization, consistent and suitable cut‐off values for the investigated population should be used. These recommendations will improve the reproducibility of studies, reduce heterogeneity, and enhance the reliability, generalizability and comparability of study results. Ultimately, they will contribute to creating more robust evidence for the potential value of BC measurements in the preoperative risk assessment of patients with CRC.

This SR and meta‐analysis has several limitations. First, we have pooled data from different populations. Thereby, we did not take into account that BC of the European population is not fully comparable with the Asian population. Second, we did not distinguish between studies based on patient characteristics, such as age, sex, location of the tumour and whether patients had undergone neoadjuvant therapy.

## Conclusions

This study created an overview of 26 different BC measurements, which were investigated for an association with a numerous amount of short‐term postoperative complications in patients undergoing CRC surgery. However, pooling data in a meta‐analysis could only be performed for VFA and overall complications and AL. Both showed higher odds for these complications if patients have more visceral fat. Although VFA appeared to be associated with overall complications and AL, the association is weak, and its clinical relevance or applicability is questionable. The current evidence is limited by methodological heterogeneity and the RoB. The studies were heterogeneous in terms of study population, BC measurements, definition of postoperative complications and statistical analysis.

Future studies should use standardized methods for measuring BC on CT scans, outcome definitions and statistical analysis. This would improve the consistency and comparability of results across studies and facilitate data pooling for meta‐analysis. Moreover, future studies should use a consistent approach to study design and analysis to reduce the potential for bias and increase the validity and reliability of findings. This would enable researchers to provide a more accurate assessment of BC, compare the results of different studies, draw more clear conclusions and facilitate the translation of research findings into clinical practice. Hopefully, this will contribute to better prediction of postoperative complications in patients undergoing colorectal surgery for CRC.

## Conflict of interest statement

The authors declare no conflicts of interest.

## Funding

This work was supported by the Dutch Research Council (Nederlandse Organisatie voor Wetenschappelijk Onderzoek [NWO]) research programme (VIDI Project Number 91719343).

## Supporting information


**Table S1.** Full Search strategy for Medline (through PubMed), Embase (via embase.com) and Web of Science (Core collection).

**Table S2.** A. Explanation of different points assessed per domain. B. Results individual assessment per domain per study.

**Table S3.** Overview of non‐significant associations.

**Table S4.** Overview of variables included in multivariable analyses.

**Figure S1.** Meta‐analysis of effect of skeletal muscle index using all cut‐off points on A) Overall complications B) Clavien‐Dindo grade 3‐5 complications.
