# Supplementary material for: Association of computed tomography‐derived body composition and complications after colorectal cancer surgery: A systematic review and meta‐analysis
Source: J Cachexia Sarcopenia Muscle. 2024 Oct 6;15(6):2234–69. doi: 10.1002/jcsm.13580 (PMC11634520; doi:10.1002/jcsm.13580)
Supplement: Supplementary file 1 — Table S1. Full Search strategy for Medline (through PubMed), Embase (via embase.com) and Web of Science (Core collection). [file JCSM-15-2234-s001.docx]

Supplementary Table 1. Full Search strategy for Medline (through PubMed), Embase (via embase.com) and Web of Science (Core collection).

**PubMed Session Results (01 Aug 2022)**

| Search | Query | Items found |
| --- | --- | --- |
| #7 | **#4 OR #6** | 996 |
| #6 | **#1 AND #2 AND #5** | 496 |
| #5 | **"Tomography, X-Ray Computed"[Mesh] OR "CT scan*"[tiab] OR "CAT scan*"[tiab] OR "computer tomograph*"[tiab] OR "computed tomograph*"[tiab] OR "computerized tomograph*"[tiab] OR "computerised tomograph*"[tiab] OR "computational tomograph*"[tiab] OR "X-ray comput*"[tiab]** | 685,699 |
| #4 | **#1 AND #2 AND #3** | 757 |
| #3 | **"Colorectal Surgery"[Mesh] OR "colorectal surg*"[tiab] OR "colon surg*"[tiab] OR "rectal surg*"[tiab] OR "proctolog*"[tiab] OR "coloproctotom*"[tiab] OR "proctocolonic"[tiab] OR (("post-operat*"[tiab] OR "postoperat*"[tiab] OR "surger*"[tiab] OR "surgical*"[tiab] OR "operat*"[tiab]) AND ("Intestine, Large"[Mesh:NoExp] OR "Cecum"[Mesh] OR "Colon"[Mesh] OR "Rectum"[Mesh] OR colorectal*[tiab] OR colon*[tiab] OR rectal*[tiab] OR rectum[tiab] OR anal[tiab] OR anus[tiab] OR appendi*[tiab] OR cecum*[tiab] OR coecum*[tiab] OR caecum*[tiab] OR cecal*[tiab] OR coecal*[tiab] OR caecal*[tiab] OR sigmoid*[tiab]))** | 164,341 |
| #2 | "**Body Composition"[Mesh] OR "body composition"[tiab] OR "body adiposity"[tiab] OR "body distribution"[tiab] OR "body fat"[tiab] OR "anthropomorphic ind*"[tiab] OR "anthropometric quantification*"[tiab] OR "Sarcopenia"[Mesh] OR sarcopen*[tiab] OR "myopeni*"[tiab] OR "Abdominal Fat"[Mesh] OR "Obesity, Abdominal"[Mesh] OR "visceral adipos*"[tiab] OR "abdominal adipos*"[tiab] OR "intra-abdominal adipos*"[tiab] OR "intraabdominal adipos*"[tiab] OR "abdominal obes*"[tiab] OR "abdominally obes*"[tiab] OR "intra-abdominal obes*"[tiab] OR "intraabdominal obes*"[tiab] OR "central obes*"[tiab] OR "baseline obes*"[tiab] OR "visceral fat*"[tiab] OR "visceral obes*"[tiab] OR "abdominal fat*"[tiab] OR "intra-abdominal fat*"[tiab] OR "intraabdominal fat*"[tiab] OR "Subcutaneous Fat"[Mesh] OR "subcutaneous fat*"[tiab] OR "sub-cutaneous fat*"[tiab] OR "subcutaneous adipos*"[tiab] OR "sub-cutaneous adipos*"[tiab] OR "panniculus adiposus"[tiab] OR "subcutaneous obes*"[tiab] OR "sub-cutaneous obes*"[tiab] OR "Muscle, Skeletal"[Mesh:NoExp] OR "skeletal musc*"[tiab] OR "muscular skeletal"[tiab] OR "muscle depletion*"[tiab] OR "muscular depletion*"[tiab] OR "myosteatos*"[tiab] OR "lean body mass*"[tiab] OR "body lean mass*"[tiab] OR "lean body weight*"[tiab] OR "lean soft tissue*"[tiab] OR "soft lean tissue*"[tiab] OR "Adipose Tissue"[Mesh:NoExp] OR "adipose tissue*"[tiab]** | 457,180 |
| #1 | **"Colorectal Neoplasms"[Mesh] OR "Cecal Neoplasms"[Mesh] OR ((neoplas*[tiab] OR tumour*[tiab] OR tumor*[tiab] OR cancer*[tiab] OR oncolog*[tiab] OR carcinom*[tiab] OR adenocar*[tiab] OR adenoma*[tiab] OR malignan*[tiab] OR precancer*[tiab]) AND ("Intestine, Large"[Mesh:NoExp] OR "Cecum"[Mesh] OR "Colon"[Mesh] OR "Rectum"[Mesh] OR colorectal*[tiab] OR colon*[tiab] OR rectal*[tiab] OR rectum[tiab] OR anal[tiab] OR anus[tiab] OR appendi*[tiab] OR cecum*[tiab] OR coecum*[tiab] OR caecum*[tiab] OR cecal*[tiab] OR coecal*[tiab] OR caecal*[tiab] OR sigmoid*[tiab]))** | 417,294 |

**Embase Session Results (01 Aug 2022)**

| Search | Query | Items found |
| --- | --- | --- |
| #8 | **#7 NOT ('conference abstract'/it OR 'conference review'/it)** | 1,293 |
| #7 | **#4 OR #6** | 2,065 |
| #6 | **#1 AND #2 AND #5** | 1,123 |
| #5 | **'computer assisted tomography'/de OR 'x-ray computed tomography'/exp OR 'colon radiography'/exp OR 'computed tomographic colonography'/exp OR 'CT scan*':ab,ti,kw OR 'CAT scan*':ab,ti,kw OR 'computer tomograph*':ab,ti,kw OR 'computed tomograph*':ab,ti,kw OR 'computerized tomograph*':ab,ti,kw OR 'computerised tomograph*':ab,ti,kw OR 'computational tomograph*':ab,ti,kw OR 'X-ray comput*':ab,ti,kw** | 1,110,420 |
| #4 | **#1 AND #2 AND #3** | 1,514 |
| #3 | **'colorectal surgery'/exp OR 'colon surgery'/exp OR 'rectum surgery'/exp OR 'proctology'/exp OR (('post-operat*':ab,ti,kw OR 'postoperat*':ab,ti,kw OR 'surger*':ab,ti,kw OR 'surgical*':ab,ti,kw OR 'operat*':ab,ti,kw) AND ('large intestine'/de OR** **'cecum'/exp OR 'colon'/exp OR 'rectum'/exp OR colorectal*:ab,ti,kw OR colon*:ab,ti,kw OR rectal*:ab,ti,kw OR rectum:ab,ti,kw OR anal:ab,ti,kw OR anus:ab,ti,kw OR appendi*:ab,ti,kw OR cecum*:ab,ti,kw OR coecum*:ab,ti,kw OR caecum*:ab,ti,kw OR cecal*:ab,ti,kw OR coecal*:ab,ti,kw OR caecal*:ab,ti,kw OR sigmoid*:ab,ti,kw))** | 307,927 |
| #2 | **'body composition'/exp OR 'body composition':ab,ti,kw OR 'body adiposity':ab,ti,kw OR 'body distribution':ab,ti,kw OR 'body fat':ab,ti,kw OR 'anthropomorphic ind*':ab,ti,kw OR 'anthropometric quantification*':ab,ti,kw OR 'sarcopenia'/exp OR sarcopen*:ab,ti,kw OR 'myopeni*':ab,ti,kw OR 'abdominal fat'/exp OR 'abdominal obesity'/exp OR 'visceral adipos*':ab,ti,kw OR 'abdominal adipos*':ab,ti,kw OR 'intra-abdominal adipos*':ab,ti,kw OR 'intraabdominal adipos*':ab,ti,kw OR 'abdominal obes*':ab,ti,kw OR 'abdominally obes*':ab,ti,kw OR 'intra-abdominal obes*':ab,ti,kw OR 'intraabdominal obes*':ab,ti,kw OR 'central obes*':ab,ti,kw OR 'baseline obes*':ab,ti,kw OR 'visceral fat*':ab,ti,kw OR 'visceral obes*':ab,ti,kw OR 'abdominal fat*':ab,ti,kw OR 'intra-abdominal fat*':ab,ti,kw OR 'intraabdominal fat*':ab,ti,kw OR 'subcutaneous fat'/exp OR 'subcutaneous fat*':ab,ti,kw OR 'sub-cutaneous fat*':ab,ti,kw OR 'subcutaneous adipos*':ab,ti,kw OR 'sub-cutaneous adipos*':ab,ti,kw OR 'panniculus adiposus':ab,ti,kw OR 'subcutaneous obes*':ab,ti,kw OR 'sub-cutaneous obes*':ab,ti,kw OR 'skeletal muscle'/de OR 'skeletal musc*':ab,ti,kw OR 'muscular skeletal':ab,ti,kw OR 'muscle depletion*':ab,ti,kw OR 'muscular depletion*':ab,ti,kw OR 'myosteatosis'/exp OR 'myosteatos*':ab,ti,kw OR 'lean body weight'/exp OR 'lean body mass*':ab,ti,kw OR 'body lean mass*':ab,ti,kw OR 'lean body weight*':ab,ti,kw OR 'lean soft tissue*':ab,ti,kw OR 'soft lean tissue*':ab,ti,kw OR 'adipose tissue'/de OR 'adipose tissue*':ab,ti,kw** | 524,563 |
| #1 | **'large intestine tumor'/exp OR ((neoplas*:ab,ti,kw OR tumour*:ab,ti,kw OR tumor*:ab,ti,kw OR cancer*:ab,ti,kw OR oncolog*:ab,ti,kw OR carcinom*:ab,ti,kw OR adenocar*:ab,ti,kw OR adenoma*:ab,ti,kw OR malignan*:ab,ti,kw OR precancer*:ab,ti,kw) AND ('large intestine'/de OR** **'cecum'/exp OR 'colon'/exp OR 'rectum'/exp OR colorectal*:ab,ti,kw OR colon*:ab,ti,kw OR rectal*:ab,ti,kw OR rectum:ab,ti,kw OR anal:ab,ti,kw OR anus:ab,ti,kw OR appendi*:ab,ti,kw OR cecum*:ab,ti,kw OR coecum*:ab,ti,kw OR caecum*:ab,ti,kw OR cecal*:ab,ti,kw OR coecal*:ab,ti,kw OR caecal*:ab,ti,kw OR sigmoid*:ab,ti,kw))** | 651,409 |

**Web of Science (Core Collection) Session Results (01 Aug 2022)**

| Search | Query | Items found |
| --- | --- | --- |
| #7 | **#4 OR #6** | 1,161 |
| #6 | **#1 AND #2 AND #5** | 520 |
| #5 | **TS=("CT scan*" OR "CAT scan*" OR "computer tomograph*" OR "computed tomograph*" OR "computerized tomograph*" OR "computerised tomograph*" OR "computational tomograph*" OR "X-ray comput*")** | 455,260 |
| #4 | **#1 AND #2 AND #3** | 918 |
| #3 | **TS=("colorectal surg*" OR "colon surg*" OR "rectal surg*" OR "proctolog*" OR "coloproctotom*" OR "proctocolonic" OR (("post-operat*" OR "postoperat*" OR "surger*" OR "surgical*" OR "operat*") AND ("colorectal*" OR "colon*" OR "rectal*" OR "rectum" OR "anal" OR "anus" OR "appendi*" OR "cecum*" OR "coecum*" OR "caecum*" OR "cecal*" OR "coecal*" OR "caecal*" OR "sigmoid*")))** | 172,082 |
| #2 | **TS=("body composition" OR "body adiposity" OR "body distribution" OR "body fat" OR "anthropomorphic ind*" OR "anthropometric quantification*" OR "sarcopen*" OR "myopeni*" OR "visceral adipos*" OR "abdominal adipos*" OR "intra-abdominal adipos*" OR "intraabdominal adipos*" OR "abdominal obes*" OR "abdominally obes*" OR "intra-abdominal obes*" OR "intraabdominal obes*" OR "central obes*" OR "baseline obes*" OR "visceral fat*" OR "visceral obes*" OR "abdominal fat*" OR "intra-abdominal fat*" OR "intraabdominal fat*" OR "subcutaneous fat*" OR "sub-cutaneous fat*" OR "subcutaneous adipos*" OR "sub-cutaneous adipos*" OR "panniculus adiposus" OR "subcutaneous obes*" OR "sub-cutaneous obes*" OR "skeletal musc*" OR "muscular skeletal" OR "muscle depletion*" OR "muscular depletion*" OR "myosteatos*" OR "lean body mass*" OR "body lean mass*" OR "lean body weight*" OR "lean soft tissue*" OR "soft lean tissue*" OR "adipose tissue*")** | 462,430 |
| #1 | **TS=(("neoplas*" OR "tumour*" OR "tumor*" OR "cancer*" OR "oncolog*" OR "carcinom*" OR "adenocar*" OR "adenoma*" OR "malignan*" OR "precancer*") AND ("colorectal*" OR "colon*" OR "rectal*" OR "rectum" OR "anal" OR "anus" OR "appendi*" OR "cecum*" OR "coecum*" OR "caecum*" OR "cecal*" OR "coecal*" OR "caecal*" OR "sigmoid*"))** | 492,443 |
