# Supplementary material for: Association of computed tomography‐derived body composition and complications after colorectal cancer surgery: A systematic review and meta‐analysis
Source: J Cachexia Sarcopenia Muscle. 2024 Oct 6;15(6):2234–69. doi: 10.1002/jcsm.13580 (PMC11634520; doi:10.1002/jcsm.13580)
Supplement: Supplementary file 2 — Table S2. A. Explanation of different points assessed per domain. B. Results individual assessment per domain per study. [file JCSM-15-2234-s003.docx]

Supplementary Table 1A. Explanation of different points assessed per domain.

| **Domain** | **Assessed points** |
| --- | --- |
| Study participation | 1. Adequate participation in the study by eligible persons 2. Description of the target population or population of interest 3. Description of the baseline study sample 4. Adequate description of the sampling frame and recruitment 5. Adequate description of the period and place of recruitment 6. Adequate description of inclusion and exclusion criteria |
| Study participation: | 1. Adequate participation in the study by eligible persons 2. Description of the target population or population of interest 3. Description of the baseline study sample 4. Adequate description of the sampling frame and recruitment 5. Adequate description of the period and place of recruitment 6. Adequate description of inclusion and exclusion criteria |
| Study attrition | Not applicable due to the study design of the studies |
| Prognostic factor measurement | 1. Adequate description of time between analyzed CT scan and surgery 2. Adequate description of who performed the analysis and if the person was blinded for outcome 3. Continuous variables are reported or appropriate cut-points (i.e. not data-dependent) are used 4. Method and setting of measurement of PF is the same for all participants 5. Appropriate methods of imputation are used for missing PF data |
| Outcome measurement | 1. A clear definition of outcome is provided 2. Method of outcome measurement in adequately valid and reliable to limit misclassification bias. 3. Method and setting of outcome measurement is same for all study participants |
| Study confounding | Not applicable due to the predictive question, as confounding is not a problem in predictive research |
| Statistical analysis and reporting | 1. There is sufficient presentation of data to assess the adequacy of the analysis 2. The strategy of model building (i.e. inclusion of variables in the statistical model) is appropriate and is based on a conceptual framework of model 3. The selected statistical model is adequate for the design of the study 4. There is no selective reporting of results |

Supplementary Table 1B. Results individual assessment per domain per study.

| **Article** | **Study participation** | **Study attrition** | **Prognostic factor measurement** | **Outcome measurement** | **Study confounding** | **Statistical analysis and reporting** |
| --- | --- | --- | --- | --- | --- | --- |
| Baastrup et al, 2020 | **Low** | **NA** | **High** | **Low** | **NA** | **Low** |
| Bachmann et al, 2018 | **Moderate** | **NA** | **Moderate** | **High** | **NA** | **Low** |
| Ballian et al, 2012 | **Moderate** | **NA** | **High** | **Moderate** | **NA** | **Low** |
| Boer et al, 2016 | **Low** | **NA** | **High** | **Moderate** | **NA** | **Low** |
| Cakir et al, 2015 | **Low** | **NA** | **High** | **Moderate** | **NA** | **Low** |
| Chai et al, 2021 | **Low** | **NA** | **High** | **Moderate** | **NA** | **Low** |
| Chen et al, 2018 | **Low** | **NA** | **Low** | **Moderate** | **NA** | **Low** |
| Der Hagopian et al, 2018 | **Low** | **NA** | **High** | **Moderate** | **NA** | **Low** |
| Dong Q et al, 2022 | **Low** | **NA** | **Low** | **Low** | **NA** | **Moderate** |
| Frostberg et al, 2021 | **Moderate** | **NA** | **Moderate** | **Moderate** | **NA** | **Low** |
| Hanaoka et al, 2017 | **Low** | **NA** | **Low** | **Low** | **NA** | **Low** |
| He et al, 2021 | **Low** | **NA** | **Moderate** | **Moderate** | **NA** | **Moderate** |
| Heus et al, 2016 | **Low** | **NA** | **Moderate** | **Moderate** | **NA** | **Low** |
| Heus et al, 2019 | **Low** | **NA** | **Moderate** | **Low** | **NA** | **Low** |
| Jochum et al, 2019 | **Low** | **NA** | **High** | **Moderate** | **NA** | **Low** |
| Kuritzkes et al, 2018 | **Low** | **NA** | **Moderate** | **High** | **NA** | **Low** |
| Lieffers et al, 2012 | **Low** | **NA** | **Moderate** | **Low** | **NA** | **Moderate** |
| Liu et al, 2019 | **Low** | **NA** | **High** | **Low** | **NA** | **Low** |
| Looijaard et al, 2019 | **Low** | **NA** | **High** | **Low** | **NA** | **Low** |
| Looijaard et al, 2020 | **Low** | **NA** | **High** | **Moderate** | **NA** | **Low** |
| Margadant et al, 2016 | **Low** | **NA** | **High** | **Moderate** | **NA** | **Low** |
| Martin et al, 2018 | **Low** | **NA** | **Low** | **Moderate** | **NA** | **Low** |
| Mauricio et al, 2018 | **Low** | **NA** | **Moderate** | **Moderate** | **NA** | **Low** |
| Mizuuchi et al, 2022 | **Low** | **NA** | **Moderate** | **Low** | **NA** | **Low** |
| Morimoto et al, 2019 | **Low** | **NA** | **High** | **Low** | **NA** | **Low** |
| Nakamura et al, 2022 | **Low** | **NA** | **Moderate** | **Low** | **NA** | **Low** |
| Nakanishi, 2018 | **Moderate** | **NA** | **High** | **Moderate** | **NA** | **Low** |
| Nattenmuller, 2019 | **Low** | **NA** | **Moderate** | **Moderate** | **NA** | **Low** |
| Okugawa et al, 2018 | **Low** | **NA** | **High** | **Low** | **NA** | **Low** |
| Olmez et al, 2021 | **Low** | **NA** | **Moderate** | **High** | **NA** | **Low** |
| Park et al, 2015 | **Low** | **NA** | **High** | **Low** | **NA** | **Low** |
| Pedrazzani et al, 2020 | **Low** | **NA** | **Low** | **Low** | **NA** | **Low** |
| Reisinger et al, 2015 | **Low** | **NA** | **High** | **Low** | **NA** | **Low** |
| Souwer et al, 2020 | **Low** | **NA** | **High** | **Moderate** | **NA** | **Low** |
| Springer et al, 2022 | **Moderate** | **NA** | **High** | **High** | **NA** | **Low** |
| Tamagawa et al, 2018 | **Low** | **NA** | **Moderate** | **Moderate** | **NA** | **Low** |
| Tankel et al, 2020 | **Low** | **NA** | **Low** | **Moderate** | **NA** | **Low** |
| Uehara et al, 2022 | **Low** | **NA** | **Moderate** | **Low** | **NA** | **Low** |
| Van der Kroft et al, 2018 | **Low** | **NA** | **Moderate** | **Moderate** | **NA** | **Low** |
| Van Vugt et al, 2018 | **Low** | **NA** | **High** | **Moderate** | **NA** | **Low** |
| Verduin et al, 2021 | **Low** | **NA** | **High** | **Low** | **NA** | **Low** |
| Watanabe et al, 2014 | **Low** | **NA** | **High** | **High** | **NA** | **Low** |
| Yang et al, 2019 | **Low** | **NA** | **Low** | **High** | **NA** | **Low** |
| Zhai et al, 2019 | **Low** | **NA** | **Low** | **Moderate** | **NA** | **moderate** |
| Zhou et al, 2020 | **Low** | **NA** | **Low** | **moderate** | **NA** | **Low** |
