# Supplementary material for: Association of computed tomography‐derived body composition and complications after colorectal cancer surgery: A systematic review and meta‐analysis
Source: J Cachexia Sarcopenia Muscle. 2024 Oct 6;15(6):2234–69. doi: 10.1002/jcsm.13580 (PMC11634520; doi:10.1002/jcsm.13580)
Supplement: Supplementary file 3 — Table S3. Overview of non‐significant associations. [file JCSM-15-2234-s002.docx]

Supplementary Table 3. Overview of non-significant associations.

| **Fat** | | | | | |  |  |  |  |  |
| --- | --- | --- | --- | --- | --- | --- | --- | --- | --- | --- |
| **Author** | **Measurement** | **Outcome** | **Univariable/**  **Multivariable** | **OR [CI]** | **P-value** |  |  |  |  |  |
| Looijaard et al. (2020)[40] | IMAT/MF  Rectus abdominis | CD3-5 | Uni | OR 1.016 [0.995-1.038] | p=0.139 |  |  |  |  |  |
|  | IMAT/MF  Rectus abdominis | CD3-5 | Multi | OR 1.012 [0.989–1.035] | p=0.302 |  |  |  |  |  |
|  | IMAT/MF psoas | CD3-5 | Uni | OR 1.113 [0.894–1.387] | p=0.339 |  |  |  |  |  |
|  | IMAT/MF psoas | CD3-5 | Multi | OR 1.133 [0.895–1.434] | p=0.298 |  |  |  |  |  |
|  | IMAT/MF back muscles | CD3-5 | Uni | OR 1.009 [0.963–1.056] | p=0.720 |  |  |  |  |  |
|  | IMAT/MF back muscles | CD3-5 | Multi | OR 1.020 [0.970–1.073] | p=0.445 |  |  |  |  |  |
| Looijaard et al. (2019)[41] | IMAT % | CD3-5 | Uni | OR 1.145 [0.843–1.557] | - |  |  |  |  |  |
|  | IMAT % | CD3-5 | Multi | OR 1.203 [0.861-1.680] | - |  |  |  |  |  |
|  | IMAT cm2 | CD3-5 | Uni | OR 1.291 [0.949–1.758] | - |  |  |  |  |  |
|  | IMAT cm2 | CD3-5 | Multi | OR 1.366 [0.980-1.902] | - |  |  |  |  |  |
| Verduin et al.(2021)[61] | MF | AL | Uni | OR 1.006 [0.963-1.052] | p=0.781 |  |  |  |  |  |
| der Hagopian et al. (2018)[29] | PRF | Intra-abdominal infection | Uni | OR 1.27 [0.5–3.2] | p>0.05 |  |  |  |  |  |
|  | PRF | AL | Uni | OR 2.59 [0.96–7.0] | p>0.05 |  |  |  |  |  |
|  | PRF | Cardiovascular complications | Uni | OR 1.53 [0.8–3.1] | p>0.05 |  |  |  |  |  |
|  | PRF | CD1 | Multi | OR 1.18 (0.59-2.35) | p>0.05 |  |  |  |  |  |
|  | PRF | CD4-5 | Multi | OR 0.73 (0.24-2.24) | p>0.05 |  |  |  |  |  |
| Frostberg et al. (2021)[31] | SFT | Any complication | Uni | OR 1.36 [0.74-2.51] | p=0.323 |  |  |  |  |  |
| Liu et al. (2019)[39] | SFT | SSI | Uni | - | p=0.31 |  |  |  |  |  |
| Verduin et al.(2021)[61] | SAT/SFA | AL | Uni | OR 1.000 [0.998–1.002] | p=0.948 |  |  |  |  |  |
| Heus et al. (2019)[34] | SAT/SFA | AL | Uni | - | p=0.173 |  |  |  |  |  |
| Kuritzkes et al. (2018)[37] | SAT/SFA | CD3-5 | Uni | - | p=0.42 |  |  |  |  |  |
| Looijaard et al. (2019)[41] | SAT/SFA | Surgical-related complications graded CD3-5 | Uni | OR 0.996 [0.711–1.394] | - |  |  |  |  |  |
| Heus et al. (2019)[34] | SAT/SFA | Ileus | Uni | - | p=0.053 |  |  |  |  |  |
|  | SAT/SFA | Urinary tract infection | Uni | - | p=0.374 |  |  |  |  |  |
|  | SAT/SFA | CD1 | Uni | - | p=0.070 |  |  |  |  |  |
|  | SAT/SFA | CD2 | Uni | - | p=0.495 |  |  |  |  |  |
|  | SAT/SFA | CD3 | Uni | - | p=0.393 |  |  |  |  |  |
|  | SAT/SFA | CD4 | Uni | - | p=0.578 |  |  |  |  |  |
| Nakamura et al. (2022)[47] | SAT/SFA | EPSBO | Uni | - | p=0.16 |  |  |  |  |  |
| Ballian et al. (2012)[24] | SAT/SFA | Complications graded by Mazeh et al. | Uni | - | p=0.17 |  |  |  |  |  |
| Heus et al. (2019)[34] | SAT/SFA | Overall complications | Multi | OR 1.001 [0.99–1.004] | p=0.314 |  |  |  |  |  |
| Kuritzkes et al. (2018)[37] | SAT/SFA | CD3-5 | Multi | OR 1.07 [0.78–1.48] | p=0.66 |  |  |  |  |  |
| Looijaard et al. (2019)[41] | SAT/SFA | CD3-5 | Multi | OR 1.139 [0.768–1.690] | - |  |  |  |  |  |
| Ballian et al. (2012)[24] | TFA/TAT | Complications graded by Mazeh et al. | Uni | - | p=0.33 |  |  |  |  |  |
| Kuritzkes et al. (2018)[37] | TFA/TAT | CD3-5 | Multi | OR 1.20 [0.94–1.51] | p=0.14 |  |  |  |  |  |
| Nattenmüller et al. (2019)[49] | TATV pelvis | Wound infection | Multi | - | - |  |  |  |  |  |
|  | TATV pelvis | Bladder dysfuction | Multi | - | - |  |  |  |  |  |
| Zhai et al. (2019)[64] | VSR | Any complication | Uni | - | p=0.253 |  |  |  |  |  |
|  | VSR | CD3-5 | Uni | - | p=1.000 |  |  |  |  |  |
|  | VSR | CD1-4 | Uni | - | p=0.459 |  |  |  |  |  |
| Ballian et al. (2012)[24] | VSR | Complications graded by Mazeh et al. | Uni | - | p=0.69 |  |  |  |  |  |
| Nattenmüller et al. (2019)[49] | VSR | Urinary tract infection | Multi | - | - |  |  |  |  |  |
| Nattenmüller et al. (2019)[49] | VFV | Burst abdomen | Multi | - | - |  |  |  |  |  |
| Park et al. (2015)[52] | VFV | CD1-2 | Uni | - | p=0.786 |  |  |  |  |  |
|  | VFV | Overall complications | Uni | - | p=0.286 |  |  |  |  |  |
| Baastrup et al. (2020)[22] | VFV | AL | Uni | - | p=0.899 |  |  |  |  |  |
|  | VFV | Any complication | Uni | - | p=0.702 |  |  |  |  |  |
|  | VFV | Any complication | Uni | OR 1.01 [0.38–2.67] | p=0.984 |  |  |  |  |  |
| Bachmann et al. (2018)[23] | VFA/VAT | Any complication | Uni | OR 1.00 | p=0.09 |  |  |  |  |  |
|  | VFA/VAT | CD3-5 | Uni | OR 1.00 | p=0.01 |  |  |  |  |  |
| Ballian et al. (2012)[24] | VFA/VAT | Complications graded by Mazeh et al. | Uni | - | p=0.97 |  |  |  |  |  |
| Zhai et al. (2019)[64] | VFA/VAT | CD1-4 complications | Uni | - | p=0.0572 |  |  |  |  |  |
|  | VFA/VAT | CD3-5 complications | Uni | - | p=1.00 |  |  |  |  |  |
| Heus et al. (2019)[34] | VFA/VAT | Ileus | Uni | - | p=0.053 |  |  |  |  |  |
|  | VFA/VAT | Urinary tract infection | Uni | - | p=0.374 |  |  |  |  |  |
|  | VFA/VAT | CD1 complications | Uni | - | p=0.070 |  |  |  |  |  |
|  | VFA/VAT | CD2 complications | Uni | - | p=0.495 |  |  |  |  |  |
|  | VFA/VAT | CD3 complications | Uni | - | p=0.393 |  |  |  |  |  |
|  | VFA/VAT | CD4 complications | Uni | - | p=0.578 |  |  |  |  |  |
| Dong et al. (2022)[30] | VFA/VAT | Gastrointestinal dysfunction | Uni | - | p=0.980 |  |  |  |  |  |
|  | VFA/VAT | Bleeding | Uni | - | p=0.777 |  |  |  |  |  |
|  | VFA/VAT | Intestinal obstruction | Uni | - | p=0.732 |  |  |  |  |  |
|  | VFA/VAT | Cardiac complications | Uni | - | p=0.984 |  |  |  |  |  |
|  | VFA/VAT | Venous thrombosis | Uni | - | p=0.973 |  |  |  |  |  |
|  | VFA/VAT | Persistant hypoalbuminemia | Uni | - | p=0.335 |  |  |  |  |  |
|  | VFA/VAT | Urinary infection | Uni | - | p=1.00 |  |  |  |  |  |
| Heus et al. (2016)[35] | VFA/VAT | Pneumonia | Uni | - | p=0.642 |  |  |  |  |  |
|  | VFA/VAT | Wound infection | Uni | - | p=0.132 |  |  |  |  |  |
|  | VFA/VAT | Urinary tract infection | Uni | - | p=0.642 |  |  |  |  |  |
| Martin et al. (2018)[43] | VFA/VAT | CD3-5 complications | Uni | 0.68 [0.32-1.45] | p=0.321 |  |  |  |  |  |
| Watanabe et al. (2014)[62] | VFA/VAT | Small bowel obstruction | Uni | - | p=0.141 |  |  |  |  |  |
| Pedrazzani et al. (2020)[53] | VFA/VAT | CD1-2 complications | Uni | - | p=0.80 |  |  |  |  |  |
|  | VFA/VAT | CD3-5 complications | Uni | - | no p-value available |  |  |  |  |  |
|  | VFA/VAT | General complications | Uni | - | p=0.87 |  |  |  |  |  |
|  | VFA/VAT | Respiratory complications | Uni | - | p=0.07 |  |  |  |  |  |
|  | VFA/VAT | Cardiac complications | Uni | - | p=0.48 |  |  |  |  |  |
|  | VFA/VAT | Surgical complications | Uni | - | p=0.48 |  |  |  |  |  |
|  | VFA/VAT | Surgical site infecition | Uni | - | p=0.33 |  |  |  |  |  |
|  | VFA/VAT | Prolonged postoperative ileus | Uni | - | p=0.13 |  |  |  |  |  |
|  | VFA/VAT | Infective complications | Uni | - | p=0.46 |  |  |  |  |  |
|  | VFA/VAT | CD≥3 | Uni | OR 0.68 [0.32-1.45] | p=0.321 |  |  |  |  |  |
| Looijaard et al. (2019)[41] | VFA/VAT | CD3-5 complications | Multi | OR 1.355 [0.996–1.845] | p>0.05 |  |  |  |  |  |
| Frostberg et al. (2021)[31] | VFA/VAT | Overall complications | Multi | OR 1.49 [0.85–2.63] | p=0.167 |  |  |  |  |  |
| Cakir et al. (2015)[26] | VFA/VAT | Pneumonia | Multi | - | - |  |  |  |  |  |
| **Muscle** | | | | | |  |  |  |  |  |
| **Author** | **Measurement** | **Outcome** | **Univariable/**  **Multivariable** | **OR [CI]** | **P-value** |  |  |  |  |  |
| Boer et al. (2016)[25] | MD TAMA L3 | Severe complications | Uni | - | p=0.454 |  |  |  |  |  |
|  | MD TAMA L4 superior | Severe complications | Uni | - | p=0.756 |  |  |  |  |  |
|  | MD TAMA L4 inferior | Severe complications | Uni | - | p=0.966 |  |  |  |  |  |
| Looijaard et al. (2020)[40] | MD rectus abdominis | Severe complications | Uni | OR 0.979 [0.951-1.008] | p=0.160 |  |  |  |  |  |
|  | MD rectus abdominis | Severe complications | Multi | OR 0.982 [0.951-1.013] | p=0.255 |  |  |  |  |  |
|  | MD back muscles | Severe complications | Uni | OR 0.985 [0.943-1.028] | p=0.489 |  |  |  |  |  |
|  | MD back muscles | Severe complications | Multi | OR 0.974 [0.930-1.021] | p=0.277 |  |  |  |  |  |
| Looijaard et al. (2019)[41] | MD/RA | CD3-5 complications | Uni | OR 0.740 [0.546–1.002] | p=0.759 |  |  |  |  |  |
| Martin et al. (2018)[43] | MD/RA | CD3-5 complications | Uni | OR 1.42 [0.87-2.31] | p=0.160 |  |  |  |  |  |
| Souwer et al. (2020)[54] | MD/RA | Any complications | Uni | OR 0.78 [0.59-1.03] | p=0.08 |  |  |  |  |  |
|  | MD/RA | Any complications | Multi | OR 0.83 [0.61-1.12] | p=0.2 |  |  |  |  |  |
|  | MD/RA | AL | Uni | OR 0.76 [0.43-1.34] | p=0.3 |  |  |  |  |  |
|  | MD/RA | AL | Multi | OR (0.90 [0.48-1.70] | p= 0.8 |  |  |  |  |  |
|  | MD/RA | Surgical complications | Uni | OR 0.81 [0.52-1.25] | p=0.3 |  |  |  |  |  |
|  | MD/RA | Surgical complications | Multi | OR 1.01 [0.71-1.44] | p=0.9 |  |  |  |  |  |
|  | MD/RA | Pulmonary complication | Multi | OR 0.87 [0.31-1.12] | p=0.1 |  |  |  |  |  |
|  | MD/RA | Severe complication | Uni | OR 0.72 [0.52-1.01] | p=0.06 |  |  |  |  |  |
|  | MD/RA | Severe complication | Multi | OR 0.79 [0.55-1.13] | p=0.2 |  |  |  |  |  |
|  | MD/RA | Mortality | Uni | OR 0.64 [0.35-1.19] | p=0.6 |  |  |  |  |  |
|  | MD/RA | Mortality | Multi | OR 0.75 [0.39-1.46] | p=0.7 |  |  |  |  |  |
| Margadant et al. (2016)[42] | MD/RA (dichotomous) | Mortality | Uni | - | p=0.162 |  |  |  |  |  |
| van der Kroft et al. (2018)[59] | MD/RA | CD2-5 | Multi | OR 0.5 [0.1-1.4] | p=0.18 |  |  |  |  |  |
| Boer et al. (2016)[25] | TPA L3 | Overall complications | Uni | - | p= 0.469 |  |  |  |  |  |
|  | TPA L4 superior | Overall complications | Uni | - | p=0.480 |  |  |  |  |  |
|  | TPA L4 inferior | Overall complications | Uni | - | p=0.881 |  |  |  |  |  |
|  | TPA L3 | CD3-5 complications | Uni | - | p= 0.320 |  |  |  |  |  |
|  | TPA L4 superior | CD3-5 complications | Uni | - | p=0.351 |  |  |  |  |  |
|  | TPA L4 inferior | CD3-5 complications | Uni | - | p=0.665 |  |  |  |  |  |
| Olmez et al. (2021)[51] | PMI | CD1-2 complications | Uni |  | p=0.896 |  |  |  |  |  |
| Springer et al. (2022)[55] | PMI | Ileus | Uni |  | p=0.10 |  |  |  |  |  |
|  | PMI | Urinary retention | Uni |  | p=0.61 |  |  |  |  |  |
|  | PMI | SSI | Uni |  | p=0.61 |  |  |  |  |  |
|  | PMI | Urinary tract infection | Uni |  | p=0.99 |  |  |  |  |  |
|  | PMI | Anastomotic leakage | Uni |  | p=0.54 |  |  |  |  |  |
|  | PMI | Pelvic abscess | Uni |  | p=0.69 |  |  |  |  |  |
|  | PMI | Renal failure | Uni |  | p=0.99 |  |  |  |  |  |
|  | PMI | Cardiovascular event | Uni |  | p=0.99 |  |  |  |  |  |
|  | PMI | Sepsis | Uni |  | p=0.99 |  |  |  |  |  |
| Tankel et al. (2020)[57] | PMI | Abdominal complications | Uni |  | p=0.265 |  |  |  |  |  |
|  | PMI | Renal complications | Uni |  | p=0.059 |  |  |  |  |  |
|  | PMI | Wound complication | Uni |  | p=0.814 |  |  |  |  |  |
| Uehara et al. (2022)[58] | PMI | CD3-5 complications | Uni |  | p=0.657 |  |  |  |  |  |
|  | PMI | Infection | Uni |  | p=0.107 |  |  |  |  |  |
|  | PMI | Surgical site infection | Uni |  | p=0.824 |  |  |  |  |  |
|  | PMI | Anastomotic leakage | Uni |  | p>0.999 |  |  |  |  |  |
|  | PMI | Wound infection | Uni |  | p=0.187 |  |  |  |  |  |
|  | PMI | Intra-abdominal abscess | Uni |  | p=0.464 |  |  |  |  |  |
|  | PMI | Urinary tract infection | Uni |  | p=0.235 |  |  |  |  |  |
|  | PMI | Pneumonia | Uni |  | p=0.187 |  |  |  |  |  |
|  | PMI | Enteritis | Uni |  | p=0.081 |  |  |  |  |  |
|  | PMI | Bowel obstruction | Uni |  | p=0.773 |  |  |  |  |  |
|  | PMI | Urination disorder | Uni |  | p=0.646 |  |  |  |  |  |
|  | PMI | Overall complications | Multi | OR 1.64 [0.79–3.41] | p=0.187 |  |  |  |  |  |
| Liu et al. (2019)[39] | RAT | SSI | Multi | - | p=0.065 |  |  |  |  |  |
| Looijaard et al. (2020)[40] | SMA rectus abdominis | CD3-5 complications | Uni | OR 0.881 [0.733–1.058] | p=0.174 |  |  |  |  |  |
|  | SMA rectus abdominis | CD3-5 complications | Multi | OR 0.931 [0.768–1.129] | p=0.466 |  |  |  |  |  |
|  | SMA lateral muscles | CD3-5 complications | Uni | OR 1.083 [0.980–1.198] | p=0.119 |  |  |  |  |  |
|  | SMA lateral muscles | CD3-5 complications | Multi | OR 1.115 [1.000–1.244] | p=0.050 |  |  |  |  |  |
|  | SMA psoas muscle | CD3-5 complications | Uni | OR 0.924 [0.793–1.077] | p=0.314 |  |  |  |  |  |
|  | SMA psoas muscle | CD3-5 complications | Multi | OR 0.920 [0.782–1.083] | p=0.316 |  |  |  |  |  |
|  | SMA back muscles | CD3-5 complications | Uni | OR 0.996 [0.909–1.092] | p=0.930 |  |  |  |  |  |
|  | SMA back muscles | CD3-5 complications | Multi | OR 0.964 [0.876–1.061] | p=0.456 |  |  |  |  |  |
| Boer et al. (2016)[25] | TAMA L3 | Overall complications | Uni | - | p= 0.312 |  |  |  |  |  |
|  | TAMA L4 superior | Overall complications | Uni | - | p=0.519 |  |  |  |  |  |
|  | TAMA L4 inferior | Overall complications | Uni | - | p=0.519 |  |  |  |  |  |
|  | TAMA L3 | CD3-5 complications | Uni | - | p= 0.589 |  |  |  |  |  |
|  | TAMA L4 superior | CD3-5 complications | Uni | - | p=0.826 |  |  |  |  |  |
|  | TAMA L4 inferior | CD3-5 complications | Uni | - | p=0.996 |  |  |  |  |  |
| Heus et al. (2019)[34] | TAMA | Overall complications | Uni | - | p=0.061 |  |  |  |  |  |
| Looijaard et al. (2019)[41] | TAMA | Surgical related CD3-5 complications | Uni | OR 1.255 [0.936-1.681] | p>0.05 |  |  |  |  |  |
|  | TAMA | Surgical related CD3-5 complications | Multi | OR 1.309 [0.942–1.819]  OR 1.315 [0.938-1.844] | p>0.05  p>0.05 |  |  |  |  |  |
| van der Kroft et al. (2018)[59] | TAMA | CD3-5 complications | Uni | - | p=0.28 |  |  |  |  |  |
| Souwer et al. (2020)[54] | SMI | Overall complications | Uni | OR 1.19 [0.91-1.57] | p=0.2 |  |  |  |  |  |
|  | SMI | Overall complications | Multi | OR 1.17 [0.87-1.57] | p=0.3 |  |  |  |  |  |
| van Vugt et al. (2018)[60] | SMI | Overall complications | Uni |  | p=0.662 |  |  |  |  |  |
| Looijaard et al. (2019)[41] | SMI | CD3-5 complications | Uni | OR 1.118 [0.833-1.502] |  |  |  |  |  |  |
|  | SMI | CD3-5 complications | Multi | OR 1.169 [0.851–1.605]  OR 1.171 [0.842-1.62] |  |  |  |  |  |  |
| Martin et al. (2018)[43] | SMI | CD3-5 complications | Uni | OR 0.63 [0.33-1.21] | p=0.162 |  |  |  |  |  |
| Reisinger et al. (2015)[3] | SMI | AL | Uni | - | p=0.13 |  |  |  |  |  |
| Souwer et al. (2020)[54] | SMI | AL | Uni | OR 0.89 [0.51-1.54] | p=0.5 |  |  |  |  |  |
|  | SMI | AL | Multi | OR 0.9 [0.48-1.69] | p=0.9 |  |  |  |  |  |
|  | SMI | Pulmonary complication | Uni | OR 0.93 [0.55-1.58] | p=0.9 |  |  |  |  |  |
|  | SMI | Pulmonary complication | Multi | OR 0.82 [0.50-1.48] | p=0.5 |  |  |  |  |  |
|  | SMI | Cardiac complications | Uni | OR 0.58 [0.31-1.07] | p=0.08 |  |  |  |  |  |
|  | SMI | Severe complication | Uni | OR 1.0 [0.72-1.38] | p=1 |  |  |  |  |  |
|  | SMI | Severe complication | Multi | OR 0.97 [0.68-1.38] | p=0.9 |  |  |  |  |  |
|  | SMI | Surgical complications | Multi | OR 1.31 [0.92-1.88] | p=0.1 |  |  |  |  |  |
| Reisinger et al. (2015)[3] | SMI | Sepsis | Uni | - | p=0.47 |  |  |  |  |  |
| van der Kroft et al. (2018)[59] | SMI | CD2-5 | Uni | OR 0.9 [0.3-2.9] | p=0.91 |  |  |  |  |  |
|  | SMI | CD2-5 | Multi | OR 0.8 [0.2-2.7] | p=0.66 |  |  |  |  |  |
| Martin et al. (2018)[43] | SMI+MD | CD3-5 | Uni | OR 0.81 [0.47-1.39] | p=0.443 |  |  |  |  |  |
| **Combination and other** |  |  |  |  |  |  |  |  |  |  |
| **Author** | | | | | | **Measurement** | **Outcome** | **Univariable/**  **Multivariable** | **OR [CI]** | **P-value** |
| Boer et al. (2016)[25] | SO TPA L3 | Overall complications | Uni | OR 0.1 [0.9–6.9] |  |  |  |  |  |  |
|  | SO TPA L4 superior | Overall complications | Uni | OR 2.6 [0.9-6.9] |  |  |  |  |  |  |
|  | SO TPA L4 inferior | Overall complications | Uni | OR 1.5 [0.6-3.7] |  |  |  |  |  |  |
|  | SO TAMA L3 | Overall complications | Uni | OR 1.3 [0.5-3.5] |  |  |  |  |  |  |
|  | SO TAMA L4 superior | Overall complications | Uni | OR 1.4 [0.5-3.8] |  |  |  |  |  |  |
|  | SO TAMA L4 inferior | Overall complications | Uni | OR 1.8 [0.7-4.8] |  |  |  |  |  |  |
|  | SO TAMA L4 superior | CD3-5 complications | Uni | OR 1.0 [0.3–3.6] |  |  |  |  |  |  |
|  | SO TAMA L4 inferior | CD3-5 complications | Uni | OR 1.2 [0.4–4.0] |  |  |  |  |  |  |
| Pedrazzani et al. (2020)[53] | SO | Mortality | Uni | - | p=0.99 |  |  |  |  |  |
|  | SO | Overall complications | Uni | - | p=0.86 |  |  |  |  |  |
|  | SO | CD3-4 | Uni | - | p=0.67 |  |  |  |  |  |
|  | SO | General complications | Uni | - | p=0.21 |  |  |  |  |  |
|  | SO | Respiratory complications | Uni | - | p=0.11 |  |  |  |  |  |
|  | SO | Surgical complications | Uni | - | p=0.92 |  |  |  |  |  |
|  | SO | AL | Uni | - | p=0.99 |  |  |  |  |  |
|  | SO | SSI | Uni | - | p=0.13 |  |  |  |  |  |
|  | SO | Infective complications | Uni | - | p=0.43 |  |  |  |  |  |
| Looijaard et al. (2019)[41] | SVR | CD3-5 | Uni | OR 0.794 [0.564-1.116] |  |  |  |  |  |  |
|  | SVR | CD3-5 | Multi | OR 0.716 [0.480–1.069] |  |  |  |  |  |  |
| Martin et al. (2018)[43] | VFA+SMI | CD3-5 | Uni | - | p=0.999 |  |  |  |  |  |
|  | VFA+MD | CD3-5 | Uni | OR 1.13 [0.65-1.95] | p=0.664 |  |  |  |  |  |
|  | VFA+SMI+MD | CD3-5 | Uni | OR 1.02 [0.41-2.57] | p=0.963 |  |  |  |  |  |
| Ballian et al. (2012)[24] | AC | Complications graded by Mazeh et al. | Uni | - | p=0.14 |  |  |  |  |  |
| Liu et al. (2019)[39] | AD | SSI | Uni | - | p=0.192 |  |  |  |  |  |
|  |  |  |  |  |  |  |  |  |  |  |

Uni=univariable; multi=multivariable; OR=odds ratio; CI=confidence interval; IMAT=intramuscular adipose tissue; MF=muscular fat; CD=Clavien Dindo classification; PRF=perirenal fat; AL=anastomotic leakage; SFT=subcutaneous fat thickness; SSI=surgical site infection; SAT=subcutaneous adipose tissue; SFA=subcutaneous fat area; EPSBO=early postoperative small bowel obstruction; TFA=total fat area; TAT=total adipose tissue; TATV=total adipose tissue volume; VSR=Visceral fat area to subcutaneous fat area ratio; VFV=visceral fat volume; VFA=visceral fat area; VAT=visceral adipose tissue; MD=muscle density; TAMA=total abdominal muscle area; L3=third lumbar vertebra; L4=fourth lumbar vertebra; RA=radiation attenuation; TPA=total psoas area; PMI=psoas muscle index; RAT=rectus abdominis thickness; SMA=skeletal muscle area; SMI=skeletal muscle index; SO=sarcopenic obesity; SVR= Skeletal muscle area to visceral fat area ratio; AC=abdominal circumference; AD=abdominal depth.
