# Supplementary material for: Association of computed tomography‐derived body composition and complications after colorectal cancer surgery: A systematic review and meta‐analysis
Source: J Cachexia Sarcopenia Muscle. 2024 Oct 6;15(6):2234–69. doi: 10.1002/jcsm.13580 (PMC11634520; doi:10.1002/jcsm.13580)
Supplement: Supplementary file 4 — Table S4. Overview of variables included in multivariable analyses. [file JCSM-15-2234-s004.docx]

Supplementary Table 4. Overview of variables included in multivariable analyses.

| **Study** | **Analyzed BC measurement** | **Outcome multivariate analysis** | **Other variables in multivariate analysis** | **Variables included based on** |
| --- | --- | --- | --- | --- |
| Baastrup et al. (2020)[22] | VFV ≥ 5.36L | Total complications | Sex, age and formation of a defunctioning stoma | Their association with development of a complication as demonstrated in the literature |
| Bachmann et al. (2018)[23] | VFA | Overall complications  CD 3-5 | Age, gender, ASA score, ERAS, MBP, surgical approach and conversion (and all other anthropometric indices) | Unclear |
| Boer et al. (2016)[25] | HU TAMA L3/L4 superior/L4 inferior  SO TPA L3/L4 superior/L4 inferior  SO TAMA L3/L4 superior/L4 inferior | Overall complications  Overall complications/Severe complications  Overall complications/Severe complications | Age, Charlson comorbidity index and previous abdominal surgery  Gender  Gender | P<0.15 in univariate analysis |
| Cakir et al. (2015)[26] | VFA (>100cm2) | Pneumonia | Age, male gender, pulmonary comorbidity | P<0.05 in univariate analysis |
| Chai et al. (2021)[27] | SMI (SA) | Overall complications (CD ≥2)  Major complications (CD ≥3) | Age, gender, BMI, CCI, ASA, American Joint Committee on Cancer stage, emergency surgery, location of cancer (colon versus rectum), SGA and albumin | Known associations in clinical practice and past research |
| Chen et al. (2018)[28] | VFA (VO) | Total complications  Surgical complications  Medical complications | Age, hypoalbuminemia, NRS, CCI, laparoscopic operation, sarcopenia and VFA/TAMA ratio  Age, BMI, Tumor location and VFA/TAMA ratio  Age, hypoalbuminemia, anemia, ASA, CCI, laparoscopic operation, sarcopenia and combined resection | P<0.10 in univariate analysis, clinically relevant |
| der Hagopian et al. (2018)[29] | PRF (≥40cm2) | Any complication (CD≥1) | Age, surgical approach, ASA and gender | Unclear |
| Dong et al. (2022)[30] | VFA (VO) | Total complications (CD≥2)  Surgical complications (CD≥2)  Medical complications (CD≥2) | Unclear | Unclear |
| Frostberg et al. (2021)[31] | VFA (VO) | Overall complications | Sex, age, cancer type, co-morbidity and surgical technique | Gender and age, which was considered important covariates for the fat distribution. The type of cancer (colon or rectal) since surgical complications may differ between the two types of cancers. Others if they demonstrated a P<0.10 in univariate analysis. |
| Hanaoka et al. (2017)[32] | MPM (mild) | Overall complications (any CD grade)  Infectious complications (any CD grade) | Age, comorbidity, tumor location, mGPS, TPA, surgical approach, and operative time  Age, mGPS and surgical approach | p <0.10 on univariate analysis |
| He et al. (2021)[33] | VSR | Overall complications | Unclear | Unclear |
| Heus et al. (2016)[35] | VFA (>100cm2)  VFA (>130cm2) | Overall complications | BMI and VFA (>130cm2)  BMI and VFA (>100cm2) | P<0.05 in the univariate analysis |
| Heus et al. (2019)[34] | VFA (>100cm2) | Overall complications | BMI, subcutaneous fat and laparoscopic surgery | P<0.05 in the univariate analysis |
| Kuritzkes et al. (2018)[37] | VFA  SFA  TFA  VTR | Major morbidity (CD≥3) | Age; gender; medical comorbidities, including hypertension, diabetes mellitus, coronary artery disease, congestive heart failure, and COPD; ASA; physical fitness grade ≥ III; American Joint Committee on Cancer (AJCC) staging; and extent of colonic resection. | P<0.01 in univariate analysis |
| Lieffers et al. (2012)[38] | SMI (SA) | Infection | Sex, cancer stage and tumour site | Unclear |
| Liu et al. (2019)[39] | RAT  AD  RATxAD | SSI | Albumin, AD and RATxAD  Albumin, RAT and RATxAD  Albumin, RAT and AD | P<0.05 in the univariate analysis |
| Looijaard et al. (2020)[40] | SM (rectus abdominis muscle, lateral muscles, psoas muscle, back muscles)  MD (rectus abdominis muscle, lateral muscles, psoas muscle, back muscles)  IMAT (rectus abdominis muscle, lateral muscles, psoas muscle, back muscles) | Severe complications (CD≥3) | Gender, age and stage of cancer. | Age (continuous variable), gender (dichotomous variable) and stage of cancer (categorical variable) as these characteristics could affect both body composition and risk of adverse outcomes. |
| Looijaard et al. (2019)[41] | MD  IMAT VAT  SAT  IMAT%  SM/VAT  SM  SMI | Severe complications (CD≥3) | Model 1: Age, stage and number of medications.  Model 1 and model 2: Age, stage, number of medications and BMI ≥30 kg/m2  Model 1 and model 2: Age, stage, number of medications and BMI ≥30 kg/m2 | Stage of disease was considered because association with cachexia and cancer-related outcomes. Number of medications was considered as a reflection of overall health and severity of the number of comorbidities that could influence both body composition and the risk of adverse outcomes. BMI ≥30 kg/m2 (model 2), to eliminate the potential influence of sarcopenic obesity. |
| Margadant et al. (2016)[42] | MD (continuous/low) | Major complications (CD≥3) | ASA and gender | Predefined potential confounders |
| Maurício et al. (2018)[44] | SMI (low) | Overall complications (CD≥2) | Cancer stage and blood transfusion | P<0.2 in the univariate analysis |
| Mizuuchi et al. (2022)[45] | PMI | AL (CD≥2) | PNI, PMI, surgical approach, left colic artery preservation, super low anterior resection and pathological T stage. | Unclear |
| Morimoto et al. (2019)[46] | VFA (VO) | POI (CD≥2) | Male gender, neoadjuvant chemotherapy, open surgery, operative time >230 minutes, estimated blood loss >50 mL and pelvic/intra‐abdominal abscess | P<0.05 in the univariate analysis |
| Nakamura et al. (2022)[47] | VSR | EPSBO | Wound infection and anastomotic leakage | P<0.05 in the univariate analysis |
| Nakanishi et al. (2018)[48] | SMI(SA) | CD≥II | Sex, history of diabetes mellitus, intraoperative blood transfusion, tumor site and operative procedure | P<0.05 in the univariate analysis |
| Nattenmüller et al. (2019)[49] | VAT (pelvis)  VAT (abdomen)/ VAT (L3/L4)  SAT (pelvis)  SAT (abdomen)  TAT (pelvis)  TAT (abdomen)  TAT (L3/L4)  VAT/SAT (L3/L4) | Surgical complications  AL  Bladder dysfunction  Burst abdomen  Cardiac complications  Bladder dysfunction  Wound infection  Burst abdomen  Medical complications  Cardiac complications  Burst abdomen  Medical complications  Wound infection  Burst abdomen  Medical complications  Cardiac complications  Bladder dysfunction  Burst abdomen  Medical complications  Cardiac complications  Burst abdomen  Cardiac complications  UTI | Age and sex | Unclear |
| Okugawa et al.(2018)[50] | Myopenia (PMI)  Myosteatosis (MD/RA) | Infectious complications  Remote infections  Infectious complications | Venous invasion, myosteatosis and blood loss. Gender, venous invasion and blood loss.  Venous invasion, myopenia and blood loss. | Previously identified confounding factors and p<0.05 in univariate analysis |
| Olmez et al. (2021)[51] | SMI (SA) | Major complications (CD≥3) | Age | P<0.05 in the univariate analysis |
| Park et al. (2015)[52] | VFV | Major complications (CD≥3)  Overall complications | Age, gender, BMI, comorbidities and location of tumor | Significant risk factors in univariate analysis |
| Pedrazzani et al. (2020)[53] | SO | Cardiac complication  PPOI | Cardiac disease, conversion and ERAS  Associated resection | P<0.05 in the univariate analysis |
| Reisinger et al. (2015)[3] | SMI (SA) | Mortality  AL  Sepsis | Sex, age, epidural analgesia, previous abdominal surgery, ASA and stage 3-4 disease.  SNAQ score 3 or more, previous abdominal surgery, tumor location, open surgery, stapled anastomosis, stage 3-4 disease and blood transfusion.  GFI score 5 or more, SNAQ score 3 or more, age, epidural analgesia and previous abdominal surgery. | P<0.05 or 0.05≤ P <0.20 in the univariate analysis |
| Souwer et al. (2020)[54] | SM  MD | Any complication  Any surgical complications  AL  Pulmonary complication  Cardiac complication  Severe complication  Mortality | Age and BMI | Consideration as confounder |
| Springer et al. (2022)[55] | PMI | Overall complications | Gender | Unclear |
| Tamagawa et al. (2018)[56] | PMI(SA) | Surgical complications |  | Unclear (backward selection) |
| Tankel et al. (2020)[57] | HUAC (SA) | Overall complications | Gender, CCI and unintended ICU admission | P<0.05 in the univariate analysis |
| Uehara et al. (2022)[58] | PMI(SA) | Overall complications  Remote infections | Sex, hemoglobin, CRP, tumor diameter, surgical approach, surgical procedure, diverting ileostomy, operating time and blood loss  Cholinesterase | P<0.05 in the univariate analysis |
| van der Kroft et al. (2018)[59] | SMI(SA)  MA | Complications (CD≥2) | Model 1: MUST and SA  Model 2: MUST and MA  Model 3: Age and SA  Model 4: Age and ASA≥3 and SA | Significant association in univariate analysis, however limited number due to statistical power, therefore several models. |
| van Vugt et al. (2018)[60] | SMI  MD | Severe complications (CD≥3) | CCI and blood loss | P<0.1 in the univariate analysis |
| Verduin et al. (2021)[61] | VFA | AL | procedure performed, ASA classification, sex, SF, BMI, age, CCI, and diabetes mellitus | Confounding factors were selected in a forward selection procedure with a limit of 5% change in effect size using a basic logistic regression model with only VF as an independent variable and AL as a dependent variable. The confounder with the largest change in effect size (at least 5%) of VF was included in the new model. The selection procedure was repeated using the remaining covariables. The procedure was stopped after none of the covariables changed the effect size by >5% or after reaching the maximum number of confounders defined as 10% of the total number of ALs. |
| Yang et al. (2019)[63] | SMI(SA) | Overall complications | Age, UICC stage II, hemoglobin, albumin, SI and ASA≥3 | P<0.1 in the univariate analysis |
| Zhai et al. (2019)[64] | VFA | Overall complications | Age and diabetes | P<0.2 in the univariate analysis |
| Zhou et al. (2020)[65] | VFA | Overall complications | NRS 2002 scores, ASA grade, CCI, metabolic syndrome, BP, TG, HDL-C, glucose, tumor location and surgical duration | Significant trend in univariate analysis |

VFV=visceral fat volume; VFA=visceral fat area; CD=Clavien Dindo classification; ASA= American Society of Anesthesiologists; ERAS=; ERAS=enhanced recovery after surgery; MBP=mechanical bowel preparation; HU=hounsfield units; TAMA=total abdominal muscle area; L3=third lumbar vertebra; L4=fourth lumbar vertebra; SO=sarcopenic obesity; TPA=total psoas area; SMI=skeletal muscle index; SA=sarcopenia; BMI=body mass index; CCI=charlson comorbidity index; SGA=subject global assessment; VO=visceral obesity; NRS=numeric rating scale; PRF=perirenal fat; MPM=Morphologic change of the psoas muscle; mGPS=modified Glasgow prognostic score; VSR=Visceral fat area to subcutaneous fat area ratio; SFA=subcutaneous fat area; TFA=total fat area; VTR=Visceral fat area to total abdominal muscle area ratio; COPD=chronic obstructive pulmonary disease; RAT=rectus abdominis thickness; AD=abdominal depth; SSI=surgical site infection; SM=skeletal muscle; MD=muscle density; IMAT=intramuscular adipose tissue; VAT=visceral adipose tissue; SAT=subcutaneous adipose tissue; PMI=psoas muscle index; AL=anastomotic leakage; PNI=prognostic nutritional index; POI=postoperative ileus; EPSBO=early postoperative small bowel obstruction; TAT=total adipose tissue; RA=radiation attenuation; PPOI=prolonged postoperative ileus; SNAQ=short nutritional assessment questionnaire; GFI=Groninger frailty index; HUAC=Hounsfield unit average calculation; ICU=intensive care unit; CRP=C-reactive protein; MA=muscle attenuation; MUST=malnutrition universal screening tool; SF=subcutaneous fat; UICC= union for international cancer control; SI=sarcopenia index; BP=blood pressure; TG=triglycerides; HDL-C=high-density lipoprotein cholesterol.
