# Supplementary material for: Association of computed tomography‐derived body composition and complications after colorectal cancer surgery: A systematic review and meta‐analysis
Source: J Cachexia Sarcopenia Muscle. 2024 Oct 6;15(6):2234–69. doi: 10.1002/jcsm.13580 (PMC11634520; doi:10.1002/jcsm.13580)

Figure S1. Meta-analysis of effect of skeletal muscle index using all cut-off points on A) Overall complications B) Clavien-Dindo grade 3-5 complications.

A.


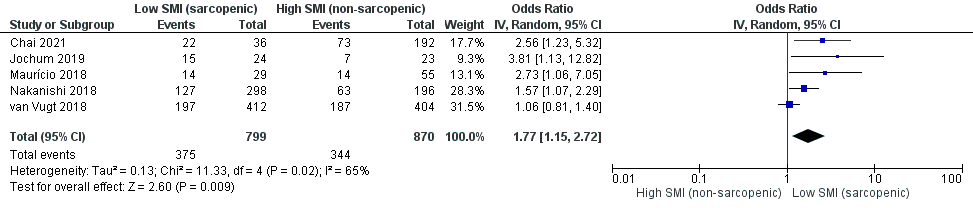


B.


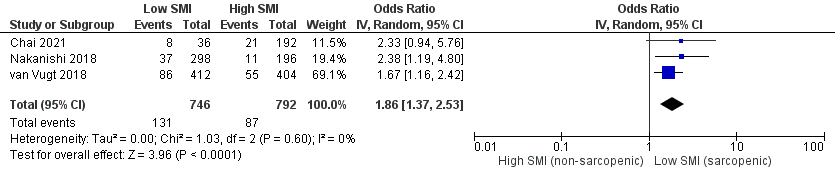

Supplement: Supplementary file 5 — Figure S1. Meta‐analysis of effect of skeletal muscle index using all cut‐off points on A) Overall complications B) Clavien‐Dindo grade 3‐5 complications. [file JCSM-15-2234-s005.docx]
